# Targeted exome sequencing identifies five novel loci at genome-wide significance for modulating antidepressant response in patients with major depressive disorder

**DOI:** 10.1038/s41398-020-0689-x

**Published:** 2020-01-23

**Authors:** Zhi Xu, Chunming Xie, Lu Xia, Yonggui Yuan, Hong Zhu, Xiaofa Huang, Caihua Li, Yu Tao, Xiaoxiao Qu, Fengyu Zhang, Zhijun Zhang

**Affiliations:** 1 The Department of Neurology and Psychiatry of Affiliated ZhongDa Hospital, and Medical School of Southeast University, 210009 Nanjing, Jiangsu China; 2 Global Clinical and Translational Research Institute, Bethesda, MD 20814 USA; 3 Center for Genetics and Genomics Analysis, Genesky Biotechnologies, Inc, 201203 Shanghai, China; 4 Genesky Diagnostics, Inc., BioBay, SIP, 215123 Jiangsu, China; 5 The Institute of Neuropsychiatry, the Key Laboratory of Development Genes and Human Diseases, the Ministry of Education and Institute of Life Sciences of Southeast University, 210096 Nanjing, Jiangsu China

**Keywords:** Depression, Pharmacogenomics

## Abstract

In order to determine the role of single nucleotide variants (SNVs) in modulating antidepressant response, we conducted a study, consisting of 929 major depressive disorder (MDD) patients, who were treated with antidepressant drugs (drug-only) or in combination with a repetitive transcranial magnetic stimulation (plus-rTMS), followed by targeted exome sequencing analysis. We found that the “plus-rTMS” patients presented a more effective response to the treatment when compared to the ‘drug-only’ group. Our data firstly demonstrated that the SNV burden had a significant impact on the antidepressant response presented in the “drug-only” group, but was limited in the “plus-rTMS” group. Further, after controlling for overall SNV burden, seven single nucleotide polymorphisms (SNPs) at five loci, *IL1A, GNA15, PPP2CB, PLA2G4C*, and *GBA*, were identified as affecting the antidepressant response at genome-wide significance (*P* < 5 × 10^−08^). Additional multiple variants achieved a level of correction for multiple testing, including *GNA11*, also shown as a strong signal for MDD risk. Our study showed some promising evidence on genetic variants that could be used as individualized therapeutic guides for MDD patients.

## Introduction

Major depressive disorder (MDD) is one of the most common severe mental disorders with a 17.8% lifetime prevalence in global population^1^. Unfortunately, current antidepressant drugs still have obvious shortcomings, including delayed onset, insufficient effectiveness, and low remission rates^2–5^, which combined with a high prevalence have significantly exacerbated the disease to be the second leading cause of disability worldwide according to the 2020 Global Burden of Disease Study^6^. Therefore, it is imperative in the study of antidepressant treatment to achieve a rapid improvement of depressive symptoms and to increase the recovery rate of the first episode of MDD^7,8^.

Pharmacogenetic studies have indicated that genomic variations are one of the critical biological factors that prominently affect the individual differences of antidepressant response, in which common single nucleotide polymorphisms (SNPs) are estimated to explain 50% or more of the variation of the antidepressant response^9^. Nevertheless, previous findings from candidate gene approaches have been poorly replicated, with insufficient predictive power to be useful in clinical practice and analytical problems of uncontrolling population stratification or genetic heterogeneity^10,11^. In addition, so far a total of eight genome-wide association studies (GWAS) of antidepressant effectiveness have been carried out mainly in Caucasian ancestry populations (eight clinical studies, ten published articles); none of these studies have found a genetic variant that achieved genome-wide significance^12–21^. However, several evaluations of the above eight clinical studies, in particular, a recent meta-analysis of Genome Based Therapeutic Drugs for Depression (GENDEP) (*n* = 738), Sequenced Treatment Alternatives to Relieve Depression (STAR*D) (*n* = 1409) and European Group for the Study of Resistant Depression (GSRD) (*n* = 1148), the three most significant pharmacogenomic studies of antidepressant responses, identified two gene sets (GO:0043949 and GO:0000183) and the integrin subunit alpha 9 (*ITGA9*) and neurexin (*NRXN3*) genes^21,22^ while the activator of transcription and developmental regulator (*AUTS2*) gene was discovered in a Korean sample^19^. However, there was no pharmacogenetic GWAS from independent Han Chinese populations. Most recently, Cai et al.^23^ have identified the association of two SNPs with the MDD risk in the most significant samples of Han Chinese, which were sirtuin 1 (*SIRT1*) gene and phospholysine phosphohistidine inorganic pyrophosphate phosphatase (*LHPP*) gene.

Exome sequencing, also known as targeted exome capture, is an efficient strategy for the selection of genomic coding sequences as well as UTR and upstream region, particularly for the study of SNPs and small insertions and deletions (indels) of known genes^24^. Thus far, only two published reports, using whole-exome sequencing, have indicated that the rs41271330 SNP of the bone morphogenetic protein 5 (*BMP5*) gene strongly associates with worse selective serotonin reuptake inhibitor (SSRI) treatment response^25^ and exm-rs1321744 located in a methylated DNA immunoprecipitation sequencing site is associated with antidepressant remission^26^.

Notably, repeated transcranial magnetic stimulation (rTMS) has been widely used in the clinical setting due to its noninvasiveness, convenience, safety, and effectiveness as an antidepressant modality^27^. It has also demonstrated a competence for rapid treatment responses, in particular, when combined with antidepressant drugs at the beginning of a medical intervention^28,29^. However, rTMS treatments for MDD still present prominently individual differences, with only about 25% response rate^30^. A few studies have found that functional serotonin 1A receptor (5-HT1A), serotonin transporter (5-HTT), and brain-derived neurotrophic factor (BDNF) gene polymorphisms affected the rTMS treatment response in MDD groups, although the results were inconsistent^31^.

In summary, genetic studies of antidepressant effectiveness with outcome follow-up at multiple time points provide a robust study design with the potential to identify factors that could inform individualized medical treatment^32^. Therefore, we conducted a targeted exome sequencing of patients undergoing antidepressant treatment for MDD, in order to identify the effect of rare and common single nucleotide variants (SNVs) on the antidepressant responses using the largest sample of untreated MDD patients in the Han Chinese population, to focus on the different effects on responses over the period of treatment and remission rates (eight weeks) and identifying genetic variants associated with the difference in response between the “drug-only” group and the “plus-rTMS” group. We identified genetic variants that could be utilized to develop individualized treatments for MDD.

## Materials and methods

### Overview

This clinical study of antidepressant effectiveness was conducted in a naturalistic clinical setting and primarily enrolled untreated adult in-patients who were experiencing current episodes of nonpsychotic unipolar depression in an ethnically homogeneous population of Han Chinese patients. Meanwhile, the study recruited well-matched healthy controls and sequenced targeted exome to analyze association with the antidepressant response.

### Subjects

The subjects enrolled in this study were Han Chinese patients referred to Zhongda Hospital MDD in-patient database. All recruited patients were between 18 and 65 years of age and met the diagnosis criteria for nonpsychotic MDD according to the Diagnostic and Statistical Manual of the American Psychiatric Association (DSM-IV; American Psychiatric Association, 1994). All subjects were newly diagnosed or recently relapsed patients, who had been drug-free for over two weeks and presented a baseline score of 18 or above on the 17-item Hamilton Depression Rating Scale (HAM-D17)^33^, having presented depressive symptoms for at least two weeks before entry. The patients were diagnosed by two independent senior psychiatrists and confirmed by a third psychiatrist if the diagnosis was inconsistent, and blind to previous evaluations.

The exclusion criteria included other documented diagnostic history on Axis 1 of the DSM-IV, independent diagnosis of personality disorder, mental retardation, pregnancy, lactation, primary organic disease, and other medical illnesses impairing psychiatric evaluation, or a history of electroconvulsive therapy within the previous six months or rTMS treatment or contraindication to rTMS. Newly diagnosed patients were also excluded if they had experienced a manic episode in the 12 months following entry. All subjects gave written informed consent for their participation in the study, which was approved by the hospital ethical committee (2016ZDSYLL100-P01), in accordance with the Declaration of Helsinki. A flowchart of the recruited subjects is shown in Fig. S1. At the end, 929 cases and 438 controls remained for the downstream analysis.

### Antidepressant treatment and outcome evaluation

All the MDD patients enrolled in this study were treated with single antidepressant drugs (drug-only) or with concurrent rTMS treatment for the first two weeks (consecutive 14 days) (plus-rTMS) according to local clinical practice. During patient treatment, selected single antidepressant drugs were administered for eight weeks and an increase in the drug dosage prescribed at baseline was allowed if the patient had not achieved “very much improved” or “much improved” symptoms based on the Clinical Global Impression (CGI) scale. Concomitant psychotropic medications were not permitted, except for a low dosage of a benzodiazepine anxiolytic (e.g. alprazolam 0.4–0.8 mg/day, estazolam 1–2 mg/day) for the alleviation of insomnia when necessary. Patients requiring a change in antidepressant drug or demonstrating nonadherence were excluded from the study.

An rTMS with an 8-shaped magnetic coil (TypeMagPro R30, MagVenture Co., Ltd; Farum, Denmark) was used and the coil was centered and fixed over the participant’s right dorsolateral prefrontal cortex (DLPFC). The conventional rTMS was administered at 80% of the motor threshold and at a low frequency of 1 Hz^34^, which remarkably reduced the attack rate of epilepsy and mania. Stimulation was given 20 min per day with each train lasting 10 min, with an interval between trains of 30 s.

The primary outcome of the study was the severity of depressive symptoms measured by the HAM-D17 symptom scale by the researchers, assessed at the baseline and at scheduled follow-up time points at weeks 2, 4, 6, and 8.

### Sample size determination and validation studies

The sample size was determined based on the repeated measures analysis of variance models^35,36^. Given that our primary goals were to identify how genetic variants affected the antidepressant response over the time of treatment, the between-subject factor in the variance model was a genotype group, which was coded as a binary in the dominant or recessive model. The calculation was performed using the PROC GLMPOWER procedure based on the Hotelling-Lawley F test with a linear exponent autoregressive correlation structure, a base correlation of 0.48, and a decay of 0.70 (see Supplementary III Methods 1 Table SM1 and SM2, Fig. SM1). The calculation of sample size showed that the “drug-only” group would be adequate at a power of 80% and alpha of 0.000006, while the number of subjects was smaller in the “plus-rTMS” group. Since the primary outcome of this study was based on the scheduled clinical evaluation at multiple time points, our findings would have provided stronger evidence than that with a population-based cross-sectional study.

### Targeted gene selection and sequencing

The selection of the targeted genes was based on pathways known to be involved in the etiology and pathophysiology of MDD and/or in mediating the antidepressant effect based on the Kyoto Encyclopedia of Genes and Genomes (KEGG) pathway. We selected various SNPs or loci that were known to be top signals in previous genetic studies of MDD and other mental disorders at the time of the study design. Altogether, we selected 1309 designable target genes for exome sequencing, with the detailed list of the genes selected for sequencing provided in the Supplementary methods (Tables SM3 and SM4). For each of these genes, both coding and noncoding regulatory regions, comprising of the five prime untranslated region (5′ UTR), 3′ UTR, and intron−exon boundaries (25 bp), were included in the sequencing targets. The sequencing of target gene was performed on an Illumina MiSeq high-throughput sequencing platform (Illumina, San Diego, CA, USA) with the assistance of Shanghai Genesky Bio-Tech Company.

### Bioinformatics analysis

#### Alignment and variant calling

Raw sequencing data were demultiplexed into individual Fastq read files with the Illumina’s Bcl2fastqv2.16.0.10 software program based on unique index pairs. Low-quality (*Q* < 15) reads/bases were trimmed using the FastX tool developed by the Hannon Lab (http://hannonlab.cshl.edu/fastx_toolkit/index.html). High-quality reads were aligned to the National Center for Biotechnology Information (NCBI) human reference genome (hg19) using the Burrows−Wheeler Aligner (BWA) software^37^. Subsequently, the aligned reads were processed further using the Picard’s MarkDuplicates, SAM tools^38^ and the Indel Realignment and Base Quality Score Recalibration platform from the Genome Analysis Toolkit^39^. The GATK v3.5^39^ and the VarScan v2.3.9 software programs^40^ were used to generate genotype information of candidate SNVs in the targeted regions for each individual, and the called SNVs were subsequently combined. A call was made to determine whether the SNVs identified had minimal depth coverage higher than (>) 20× and a quality score >30 in more than 80% of the subjects sequenced. After in-house quality control for both clinical and sequencing data, a total of 126,316 SNVs within 1309 genes, comprising 17,870 common SNPs and 108,173 rare SNVs (minor allele frequency [MAF] <0.01), of which 107,875 rare SNVs were successfully annotated.

#### SNVs annotations

The ANNOVAR software program (released on February 1, 2016) was used for the annotation of SNVs^41^. SNVs were divided into two groups: SNPs and rare variants. Any SNVs with a minor allele frequency of ≤1% and with a genotyping rate of more than 90% was considered a SNP (SNPs that failed the Hardy-Weinberg equilibrium test at a significance level of 0.00001 were removed). Similarly, SNVs presenting a minor allele frequency of <1% were considered rare variants. In addition, we created a priority score for each rare variant by integrating multiple annotation information, such as conservation, frequency, and protein scores (e.g., PolyPhen). A detailed description of the level-score can be found in Table SM5 (Supplementary Methods).

We then performed an independent validation of the top SNPs that may affect the antidepressant response, using improved multiplex ligation detection reactions (iMLDR)^42^. The SNPs presenting a 98% accuracy of the genotypes between the raw and validation data were reported here (Supplementary Methods).

### Statistical analysis

Common and rare SNVs were separated for further data processing and quality control before subsequent analysis. Rare SNVs (MAF < 1%) were further classified by gene region and function class. For statistical analysis purposes, we also classified the SNVs by comparing them with existing databases, such as the 1000 Genomes, and were consistently called by two computer programs. A sum of the number of minor alleles, called the SNV burden, was obtained for each MDD patient, in order to assess the influence on the antidepressant treatment response. After quality controls were performed, we utilized all common variants for the examination of cryptic relatedness and the control of population stratification. Identity by descent (IBD) analysis of common SNPs was used to remove individuals with cryptic relatedness (estimated kinship coefficient > 0.25) and multidimensional scaling (MDS) components were used for the examination of genetic outliers or population structure. The number of common SNPs in this study was adequate to reveal the population structure^43,44^. The PLINK v1.9 computer software was used for this analysis.

The analysis of the primary outcome was measured by the HAM-D17 assessment scale, which was first performed on the overall SNV burden and then by gene region and function class for the antidepressant response. After controlling for the global SNV burden (log-transformed), we then examined the common SNPs for the antidepressant response. A general linear model with random effect was used to analyze the primary outcome (HAM-D17)^45^, which were log-transformed to be close to the normal distribution and evaluated at multiple time points during the clinical study. The main effect was common genetic variants and genotypes by time interaction, while adjusting for covariates of sex, age of onset, number of depressive episodes, duration of illness (months), level of education, type of medication, and first MDS components. All analysis was performed using the PLINK v1.9, SAS release 9.4 and R3.3.2 release software platform (https://www.r-project.org/).

Furthermore, three levels of threshold were defined for significance or as a strong signal. While our study was based on targeted exome sequencing analysis, we still applied a standard of genome-wide significance to assure a robust finding (*P* < 5 × 10^−08^). We have also tried to present all possible top SNPs that achieved a Bonferroni correction (*α* = 9.7 × 10^−07^) and survived the correction for the number of SNPs (*α* = 0.05/12,561 = 3.98 × 10^−06^) for further testing of these top molecular signals.

### *cis*-eQTL analysis, RNA expression, and bioinformatics investigation

Multiple datasets of RNA expression in human tissues were used to validate our top findings at the level of gene or protein expression and *cis*-regulatory associations. They were shown as follows: (1) the RNA-seq data provided by the HPA (https://www.proteinatlas.org/); (2) the RNA-seq data of the GTEx (https://www.gtexportal.org), which was mapped using the Ensembl gene ID and with *the cis*-eQTL analysis carried out online; and (3) a microarray RNA expression of postmortem human brains was analyzed for *cis*-regulatory effects of the rs8092 SNP on the *GNA15* locus, which was based on 88 subjects of European ancestry (for consistency with the other samples), older than five years of age (http://braincloud.jhmi.edu/)^46^. Unless specified, all LD were calculated using the sample of the Han Chinese in Beijing, China (CHB)-Japanese in Tokyo, Japan (JPT) in the 1000 Genomes (*N* = 404).

## Results

### Clinical study for the effectiveness of antidepressant intervention

Finally, a total of 929 patients with MDD enrolled in this study (drug-only 530 vs. plus-rTMS 399). The single antidepressant drug includes SSRIs (51%), serotonin-norepinephrine reuptake inhibitors (SNRIs; 24%), and other antidepressant drugs (25%). As shown in Table 1 and Tables S1−S2, there were no significant differences (*P* > 0.05) in the mean or frequency of sex, family history, type of drugs, duration of illness, and the baseline clinical symptom, which was a log-transformed HAM-D17 (all *P* > 0.05). We noted a difference (*P* < 0.05) in the number of depressive episodes, age, education and age of onset between the two treatment groups at baseline. However, in a multiple regression model analysis, only the level of education (*P* = 0.0472) and age of onset (*P* = 0.0346) were slightly different between the two treatment groups.

**Table 1:** Summary of the demographics, covariates, and clinical symptoms statistics by treatment group.

		Drug-only (*N* = 530)			plus-rTMS (*N* = 399)			
		*N*	%/Mean	SD	*N*	%/Mean	SD	*P*
Categorical variables								
Sex								
	Male	176	0.335		127	0.318		0.60
	Female	350	0.665		272	0.682		
Number of episode								
	≤2	395	0.745		339	0.850		0.0001
	3	65	0.123		38	0.095		
	≥4	70	0.132		22	0.055		
Level of education								
	Less than high school	106	0.200		110	0.276		0.021
	High school	315	0.594		208	0.521		
	More than high school	109	0.206		81	0.203		
Family history	None	475	0.896		360	0.902		0.76
	Yes	55	0.104		39	0.098		
Type of drug								
	SNRI	118	0.223		100	0.251		0.23
	SSRI	272	0.513		182	0.456		
	Others	140	0.264		117	0.293		
Continuous variables								
	Age of onset (years)	530	44.82	14.42	399	42.62	14.65	0.023
	Duration of illness (months)	530	55.61	82.68	399	45.75	72.39	0.058
	Number of episode	530	2.07	2.05	399	1.59	0.98	<0.0001
	Age (years)		49.20	14.36		46.29	14.48	0.0024
HAM-D17 score								
	Baseline	530	23.94	4.97	399	23.24	3.97	0.021*
	Week 2	530	13.27	6.08	399	10.57	4.52	
	Week 4	530	9.38	5.15	397	6.90	3.68	
	Week 6	528	7.39	4.80	394	5.61	3.82	
	Week 8	510	6.52	4.58	366	5.56	4.52	
Log HAM-D17								
	Baseline	530	3.16	0.20	399	3.13	0.17	0.059*
	Week 2	530	2.47	0.49	399	2.25	0.49	
	Week 4	530	2.09	0.57	397	1.79	0.56	
	Week 6	528	1.78	0.69	394	1.50	0.69	
	Week 8	510	1.62	0.75	366	1.43	0.78	

*P value for a test indicating the differences in HAM-D17 or log-HAM-D17 at baseline between the two treatments.

There were no significant differences in the baseline log-HAM-D17 score between the two groups (*P* = 0.059). However, more patients in the “plus-rTMS” group presented two or fewer depressive episodes (*P* = 0.0001) (Table 1). Obviously, patients in the “plus-rTMS” group had more effective response (58.65% vs. 40.76%) within the first two weeks (*P* = 0.00014) and four weeks (*P* = 0.0054) after treatment as compared with the “drug-only” group. Furthermore, the overall remission rate in the “plus-rTMS” group was 65.20%, higher than the 55.66% of “drug-only” group (*P* = 0.0035) (Table 2).

**Table 2:** The rate of effective responses to antidepressant treatments at multiple time points during the duration of the clinical study and the remission at the end of the trial in all patients, as well as in the different treatment groups.

Effective response	Overall		Drug-only		Plus-rTMS		*P*
	*N* = 929	%	*N* = 530	%	*N* = 399	%	
Baseline							
Week 2	450	48.44	216	40.76	234	58.65	0.00014
Week 4	738	79.44	383	72.26	355	88.97	0.0054
Week 6	810	87.19	444	83.77	366	91.73	0.211
Week 8	780	83.96	446	84.15	334	83.71	0.972
Remission rate	555	59.74	295	55.66	260	65.20	0.0035

An effective response was defined as a ratio of the improvement in the HAM-D17 scale from the baseline, to the follow-up time point divided by the HAM-D17 score at baseline, greater than 50%. Remission was defined as an HAM-D17 score of < 7 at the end of the clinical study.

### The SNV burden modulates the antidepressant response of MDD patients depending on the specific treatment and gene region

A total of 107,875 rare SNVs were found located mainly in intronic, exonic, and UTR3 variant regions (Table S3). Approximately half of the rare SNVs (56,552) found were detected in patients with MDD and not in the other 1000Genomes or the 438 healthy controls. Moreover, out of the total SNVs found, 53.61% of the rare SNVs (57,830) were consistently detected with both the GATK and the Var Scan variant-calling software applications. In addition, exonic variants increased from 24.2 to 36.2% and UTR3 variants increased from 22.7 to 28.99%, while intronic variants decreased (Table S4).

**Fig. 1:**
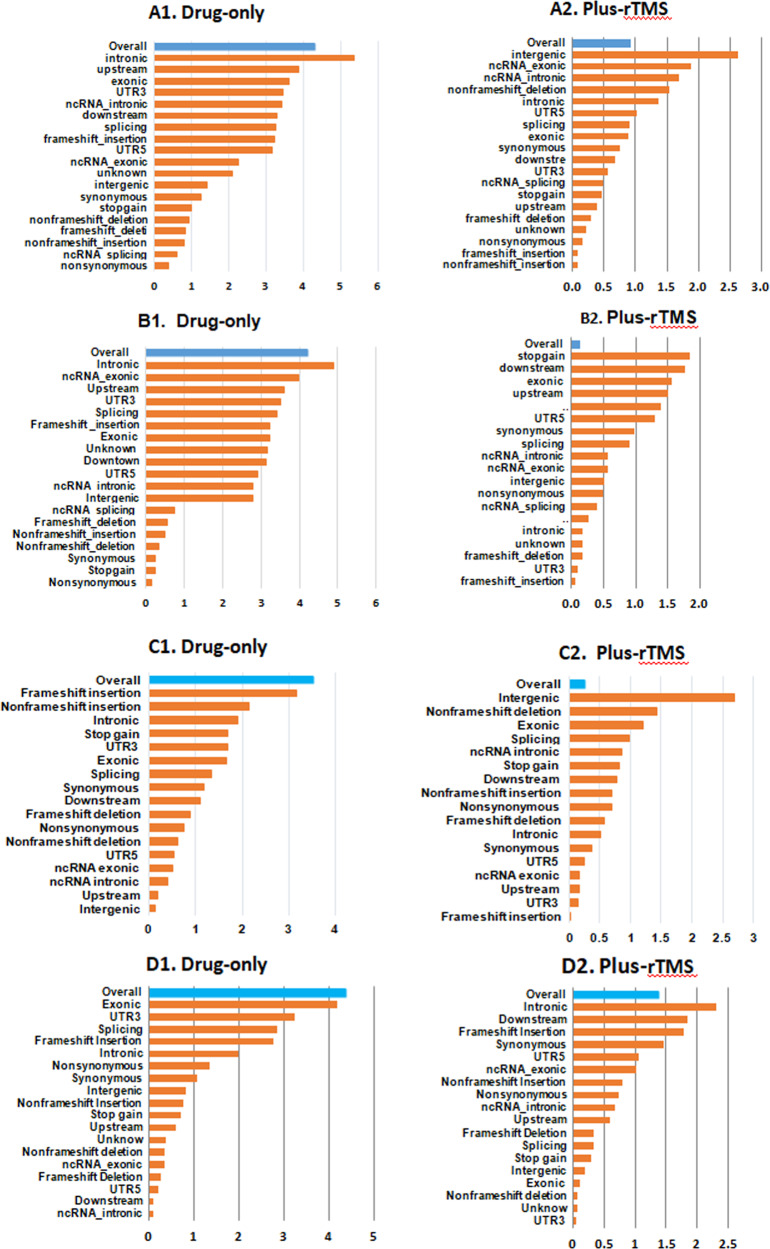
The single nucleotide variant (SNV) burden by gene region and functional class on the antidepressant response presented in each treatment group. **a** The burden of all 107,875 SNVs: A1 the “drug-only” group and A2 the “plus- repetitive transcranial magnetic stimulation (rTMS)” treatment group. **b** The burden of the 56,552 SNVs specifically detected in major depressive disorder (MDD) patients, but not found in either the 1000Genomes or healthy control subjects: **B1** the “drug-only” group and **B2** the “plus-rTMS” treatment group. **c** The burden of all 57,830 SNVs consistently called using the GATK and VarScan variant software programs: C1 the “drug-only” group and C2 the “plus-rTMS” treatment group. **d** The burden of the 27,220 SNVs detected specifically in MDD patients, but not in either the 1000Genomes or healthy control subjects used for this study, which were consistently called by the GATK and VarScan variant software programs: D1 the “drug-only” group and D2 the “plus-rTMS” treatment group (*x*-axis is the negative logarithm of *P* value).

Interestingly, the SNV burden, analyzed by various methods (as shown in Tables S5−S8 and Fig. 1 A1−A2 and B1−B2), was found to modulate the antidepressant response only in the “drug-only” group. Specifically, the antidepressant response was affected by the SNV burden at intronic, upstream, exonic, UTR3, ncRNA intronic, downstream, and splicing regions (*P* ≤ 5.32 × 10^−04^) in the “drug-only” group. In contrast, only intergenic SNVs showed a signal in the “plus-rTMS” treatment group (*P* = 2.37 × 10^−03^) (Table S6). Notably, MDD patients with a higher level of SNV burden tended to exhibit a slower response to antidepressants only in the “drug-only” group (*P* = 1.23 × 10^−04^) (Table S9).

Similarly, the SNV burden was also found to modulate the antidepressant response differently by treatment group when our analysis was focused on the 57,830 SNVs that were consistently called by the GATK and VarScan programs, in which the SNV burden markedly affected the antidepressant response only in the “drug-only” group as compared to the “plus-rTMS” group (see Tables S10−S13, Fig. 1 C1−C2 and D1−D2 for detail).

### Common variants modulate the antidepressant response

Controlling for the SNV burden, we further performed an analysis of 12,561 SNPs under a dominant and recessive model to search for common variants that may modulate the antidepressant response differently by treatment group (Figs. S2, S3). To avoid too fewer number of homozygotes, this analysis only considered SNPs with MAF > 5%. (1) Importantly, the seven SNPs at five loci were found associated with the antidepressant response at genome-wide significance (5 × 10^−08^) (Table 3). These included SNPs rs3783553 and rs3783550 at the *IL1A* loci (minimum *P* = 1.41x10^−08^), rs11671393 at *GNA15* (*P* = 4.95 x 10^−12^), and rs4733201 at *PPP2CB* (*P* = 1.07 × 10^−08^), which were identified in the “drug-only” group, and SNPs rs2303744 at *PLA2G4C* (minimum *P* = 2.70 × 10^−10^), rs9628662 (*P* = 2.45 × 10^−09^), and rs12034326 (*P* *=* 4.4 × 10^−08^) at *GBA*, which were identified in the “plus-rTMS” group. The detailed estimates of treatment responses by the SNP genotype and treatment time indicated that the trend of the responses was different between the genotype groups (Table S14). (2) In addition, we observed ten SNPs, including *GNA11* with strong signals for the antidepressant response in the “drug-only” or the “plus-rTMS” group, respectively, achieved a strict Bonferroni correction (*α* = 9.9 × 10^−07^) (see Table 3 for detail). (3) Additional SNPs at loci *AGXT*, *ACE*, *CYP2C8*, *SGMS1, PLCH2*, *RPTOR*, and *GNA11* also achieved a level of threshold for correcting the number of SNPs tested (*α* = 0.05/12,561 = 3.98 × 10^−06^) (Table 3).

**Table 3:** Top SNPs showing a genome-wide significance or a strong signal for modulating the antidepressant response (*P* *<* 9 × 10^−07^; Bonferroni correction for the number of SNPs tested as well as the genetic model and stratification by treatment)*.

Chr	SNP	BP	*Gene*	Location	A1	A2	Treatment response				
							NDF	DDF	F	*P*	
Drug-only group											
Dominant model											
2	rs3783553	113,531,715	*IL1A*	UTR3	TGAA	-	4	2047	10.69	1.4E−08**	
2	rs3783550	113,532,885	*IL1A*	Intronic	T	G	4	2051	9.97	5.4E−08**	
1	rs3817192	204,400,650	*PIK3C2B*	Intronic	C	A	4	1996	8.60	7.0E−07*	
2	rs1609682	113,540,205	*IL1A*	Intronic	T	G	4	2047	8.30	1.2E−06	
2	rs12695032	241,815,473	*AGXT*	Intronic	A	G	4	2048	7.98	2.2E−06	
17	rs183190490	61,557,305	*ACE*	Intronic	A	G	4	2025	7.87	2.7E−06	
10	rs2071426	96,828,323	*CYP2C8*	Splicing	C	T	4	2031	7.79	3.2E−06	
Recessive model											
19	rs11671393	3,151,676	*GNA15*	ncRNA_intron	C	G	4	2048	14.93	5.0E−12**	
8	rs4733201	30,670,390	*PPP2CB*	Upstream	T	G	4	2048	10.84	1.1E−08**	
4	rs3749525	66,270,241	*EPHA5*	Intronic	G	A	4	2048	8.59	7.1E−07*	
10	rs11006229	52,350,006	*SGMS1*	UTR5	T	C	4	2048	8.17	1.6E−06	
10	rs1810576	52,220,420	*SGMS1*	Splicing	C	T	4	2048	8.17	1.6E−06	
Plus-rTMS group											
Dominant model											
1	rs78810070	57,176,393	*PRKAA2*	UTR3	C	T	4	1495	7.99	2.3E−06	
1	rs10797428	2,411,451	*PLCH2*	Intronic	A	G	4	1531	7.92	2.6E−06	
1	rs12727342	2,409,892	*PLCH2*	Intronic	A	G	4	1520	7.86	2.8E−06	
Recessive model											
19	rs2303744	48,602,948	*PLA2G4C*	Exonic	T	C	4	1535	12.85	2.7E−10**	
1	rs9628662	155,206,341	*GBA*	Intronic	T	G	4	1535	11.67	2.5E−09**	
1	rs12034326	155,214,473	*GBA*	UTR5	A	G	4	1535	10.12	4.4E−08**	
19	rs1653554	48,608,472	*PLA2G4C*	Intronic	G	A	4	1535	8.79	5.1E−07*	
2	rs7557421	46,207,618	*PRKCE*	Intronic	A	G	4	1535	8.70	6.0E−07*	
17	rs9911574	78,795,868	*RPTOR*	Intronic	G	A	4	1535	7.94	2.5E−06	
19	rs8092	3,123,635	*GNA11*	UTR3	T	C	4	1535	7.87	2.8E−06	

“Drug-only” patients treated with pharmacological agents, and “plus-rTMS” patients treated with pharmacological agents plus rTMS for the first two weeks of the study.

Only SNPs with minor allele frequencies >5% were included for analysis.

The *P* value indicates the interaction between the time of treatment and the genotype from a general linear random-effect model perspective (coded as a binary variable under a dominant model or a recessive model for convenient interpretation of findings).

**SNPs achieving genome-wide significance; *SNPs with Bonferroni corrections (*α* = 9.9 × 10^−07^).

### Common variants associated with both antidepressant response and risk of MDD

**Fig. 2:**
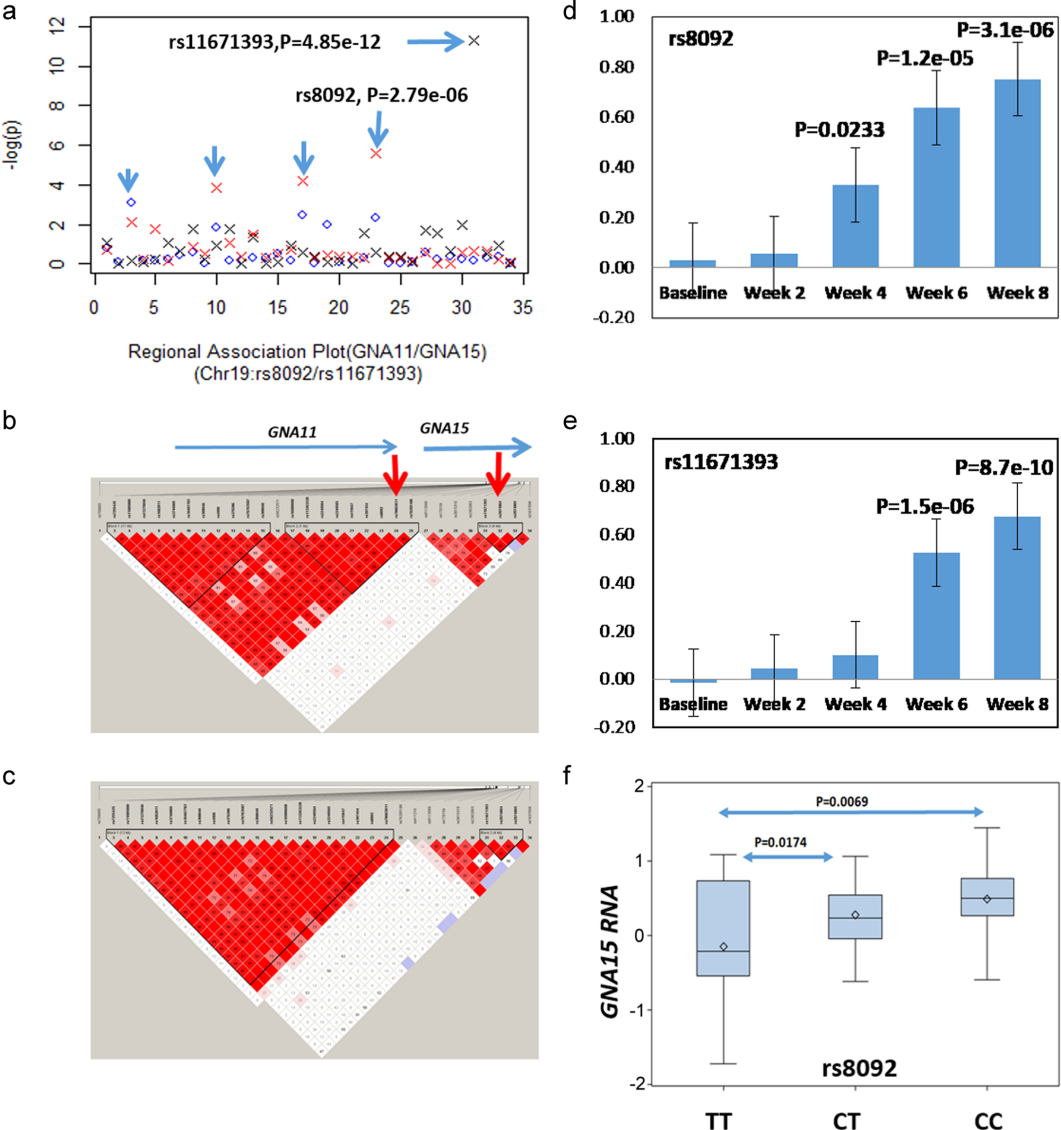
Genetic variants at the *GNA11−GNA15* loci significantly affect the antidepressant response. **a** Scatter plots depicting the *P* values obtained for the antidepressant response (X sign, a dot indicates a *P* value for the MDD risk association). **b** A linkage disequilibrium (LD) plot of MDD patients (based on common variants from the sequencing analysis). **c** An LD plot of healthy control subjects. **d** Differences in the log of the Hamilton Rating Scale for Depression-17-Item (HAM-D17) scale ratings between carriers of the major risk allele C (REC = 0) and homozygous for the minor T allele of the rs8092 single nucleotide polymorphism (SNP) at the *GNA11* locus in the “plus-rTMS” treatment group. **e** Differences in the log-HAM-D17 between carriers of the major allele G (REC = 0) and homozygous for the minor allele of the rs1167393 SNP at the *GNA15* locus in the “drug-only” group. **f** The major risk allele C of the rs8092 SNP was associated with the expression of *GNA15* in postmortem human brains of adult subjects of European ancestry (*n* = 88, subjects five years of age or more, *P*_ANOVA = 0.0006) adjusted for RIN, postmortem interval (PMI), age, sex, and pH. The plot was made based on post hoc analysis of the least square (LS)-means in the RNA expression of *GNA15*.

Interestingly, only the rs8092 SNP at the UTR3 variant of the *GNA11* loci showed the association with both antidepressant response and MDD risk (Table S15). This SNP is also upstream of *GNA15* where the rs11671393 SNP showed genome-wide significance (*P* = 4.85 × 10^−12^) for the antidepressant response (Fig. 2a). These two SNPs were less likely in linkage disequilibrium (LD; *R*^2^ = 0.0144 and *D*′ = 0.1215) (Fig. 2b, c). Patient carriers of the major risk allele C of rs8092 (Fig. 2d) and allele G of rs11671393 at *GNA15* tend to have a slower and poorer response to the antidepressant treatment (Fig. 2e). In addition, the rs8092 SNP had a *cis*-association with *GNA15* in the prefrontal cortex of postmortem human brains (*N* = 88, *P* = 7.0 × 10^−03^ (Fig. 2f) and in other tissues (see Fig. S4). In addition, significant *cis*-eQTL (expression quantitative trait locus) in human tissue samples of the genotype-tissue expression (GTEx) dataset was found for all five loci involved in the antidepressant response at genome-wide significance (Table S16), which were significant after multiple testing correction (FDR < 0.05).

### Replication of loci reported in previous genome-wide association studies of MDD and genetic studies of the antidepressant response

Despite the different convergence of individual SNPs between the various genotyping platforms, in this study we made an effort to validate the loci reported in previous GWAS of MDD and genetic studies of the antidepressant response. Out of the seven loci reported in previous GWAS of MDD^23^^,47–49^ included in our study, seven SNPs at *ENOX1, ESR2, PMFBP1*, and *EP300* showed a significant impact on the antidepressant response in the “drug-only” group (*P* < 0.05), whereas the rs730210 SNP at *ENOX1* and the rs3818121 SNP at *EP300* showed a significant impact on the antidepressant response in the “plus rTMS” group (*P* < 0.05).

Furthermore, out of the suggested loci found to be promising candidates for the antidepressant response in clinical applications after validation^34^, multiple SNPs at *ABCB1, BDNF, CYP2C19, SLC6A2, CYP2D6*, and *HTR2A* showed a trend for influencing antidepressant response at a nominal significance in our study (*P* < 0.05) (Table S17). However, we did not find a positive validation for SNPs at the *GNB3* and *FKBP5* loci, although they were included in the present study. Unfortunately, *ITGA9* and *NRXN3* were not included in this study, and we were not able to replicate any SNPs at loci *AUTS2* (*P* > 0.05).

## Discussion

The present study firstly found significant differences in the rate of response to antidepressant treatment after two weeks, with a remission rate after eight weeks treatment between the two subgroups, in which the “plus-rTMS”-treated patients presented a more effective response to treatment as compared to the “drug-only” group, indicating that the concurrent use of rTMS may have an additional rapid improvement in the clinical symptoms.

It is interesting to note that the SNV burden had a significant impact on the antidepressant response primarily in the “drug-only” patients. This could be due to the fact that these patients had more repeated depressive episodes than those receiving TMS, perhaps a consequence of a higher level of SNV burden which may in turn relate to a higher likelihood of having treatment-resistant depression^50^. On the other hand, the patients in the “plus-rTMS” group presented higher rates of effective responses after being treated for two weeks, which is consistent with previous findings^51^. Thus, the additional treatment effect may, to some extent, overcome the burden imparted by these SNVs.

Importantly, our study had identified the seven SNPs (MAF > 5%) at the five loci *IL1A*, *GNA15*, *PPP2CB*, *PLA2G4C* and *GBA* for treatment response at genome-wide significance in MDD patients. Furthermore, we had identified the additional ten SNPs including rs8092 at *GNA11* achieved a threshold for multiple testing correction. Importantly, the SNPs identified here were strongly associated with an increased likelihood of remission at the end of the clinical study for MDD patients.

Some of the above seven SNPs were randomly identified or in LD with other SNPs, noted for their strong signals, but none of these SNPs had been shown to reach genome-wide significance in previous GWAS of related phenotypes. For example, the 4-bp indel at rs3783553 and rs3783550 SNP at *IL1A* identified in this study have previously been reported to be associated with various cancers and severe inflammatory diseases, such as ankylosing spondylitis mostly in Asian populations, including Chinese Han^52,53^. The rs3783550 SNP at *IL1A* was also found to be in perfect LD (*R*^2^ = 0.197 and *D*′ = 1) with the rs11677416 SNP at *CKAP2L-IL1A*, which has been found to have a strong signal for antipsychotic response in patients with schizophrenia treated with olanzapine^54^. The rs11671393 SNP at *GNA15* was also found to be in perfect LD with the rs2302062 and rs11890198 SNPs, previously noted to have strong signals for glucose homeostasis^55^ and heart failure^56^. In addition, one variant (chr8:30765196-T) at the *PPP2CB* loci had been noted as a signal for daytime sleepiness^57^. However, rs1549637 SNP in *PLA2G4C* is reportedly related to negative symptom severity in schizophrenic patients^58^, and the rs251684 variant of *PLA2G4C* is associated with autism spectrum disorder^59^. Notably, multiple variants at the *GBA* locus have been found associated with Parkinson’s disease in four previous GWAS^60–63^.

In addition, all the seven SNPs found here were eQTLs in human tissues. For instance, the *IL1A* encodes the interleukin 1 alpha (IL-1α) protein, also known as hematopoietin 1, which is a pleiotropic cytokine involved in various immune responses, inflammatory processes, and hematopoiesis^64^. *GNA15*, which encodes the G protein subunit alpha 15, is highly expressed in esophageal tissues and bone marrow in the GTEx dataset (https://www.gtexportal.org). *PPP2CB*, which encodes the phosphatase two catalytic subunit beta enzyme, is highly expressed in the brain and by the immune system, as shown in the HPA dataset. Furthermore, the *PLA2G4C* locus encodes the protein phospholipase A2 group IVC, which is highly expressed in multiple human tissues, including the brain, the gastrointestinal tract, the bone marrow, and the immune system. The *GBA* encodes the enzyme glucosylceramidase beta, which is highly expressed in human tissues, including the brain, the immune system, and the gastrointestinal tract. Similarly, the rs12034326 SNP at the *GBA* locus, demonstrated a strong *cis*-association in multiple tissues including the esophagus.

Interestingly, one of the SNPs achieving significance following correction for multiple testing—rs8092 SNP at *GNA11*—is also associated with both the antidepressant response and risk of MDD. First, rs8092 was shown to have a strong signal for the antidepressant response close to the Bonferroni correction. Additionally, it presented the strongest signal for the MDD association among all the top SNPs identified for the antidepressant response. While the association of rs8092 with MDD was not found at genome-wide significance in our study, rs8092 has been previously identified at genome-wide significance analysis for multiple sclerosis, in which depression is a critical complication, as shown in a study of a large homogenous Scandinavian cohort through identity by descent (IBD) mapping^65^. Further, *GNA11* (*P* < 0.0054) was implicated in association with MDD by a pathway analysis study from a previous GWAS of MDD of European ancestry^66^. Notably, the rs8092 SNP was previously shown to have a *cis*-association with *GNA11* in human brains from the GTEx dataset and in the prefrontal cortex of postmortem human brains, together with a nearby gene *GNA15*, in which the rs11671393 SNP was found to be associated with antidepressant response at genome-wide significance in our study. Similarly, mutations in the *GNA11* locus have been found associated with hypocalciuric hypercalcemia type II (HHC2), hypocalcemia dominant 2 (HYPOC2), and uveal melanoma conditions^67^^,68^. All of this evidence may support the notion that the rs8092 SNP is a candidate for both the antidepressant response and risk of MDD.

There are several limitations to this study. One of its weaknesses is the lack of a replication cohort. The study relied on the self-reporting of several factors including family history, which may be under-reported, although we found this factor to have no influence on the results. In assessing the exome, we may not identify all possible genetic factors, some of which may reside in nonexomic—intergenic or intronic—regulatory sequences.

In summary, our study shows that both rare and common genetic variants can modulate the antidepressant response differently between the two subgroups of antidepressant treatments selected here. These findings generate promising evidence for the use of individualized and precision medicine in patients with MDD and may provide new insights into the underlying molecular mechanism of antidepressant response.

## Supplementary information

Supplementary materials
