# Supplementary material for: Targeted exome sequencing identifies five novel loci at genome-wide significance for modulating antidepressant response in patients with major depressive disorder
Source: Transl Psychiatry. 2020 Jan 23;10:30. doi: 10.1038/s41398-020-0689-x (PMC7026085; doi:10.1038/s41398-020-0689-x)
Supplement: Supplementary file 1 — Supplementary materials [file 41398_2020_689_MOESM1_ESM.docx]

**Supplementary Information**

**Targeted exome-sequencing identifies five novel loci at genome-wide significance for modulating antidepressant response in patients with major depressive disorder**

**Zhi Xu^1^, Chunming Xie^1^, Lu Xia^2^, Yonggui Yuan^1^, Hong Zhu^1^, Xiaofa Huang^1^, Caihua Li^3^, Yu Tao^3^, Xiaoxiao Qu^4^, Fengyu Zhang^2#^, Zhijun Zhang^1, 2, 5,#^**

1. Supplementary tables
2. Supplementary figures
3. Supplementary methods
4. **Supplementary tables**

**Tables S1.** Summary statistics of primary outcome in all patients and by treatment group

|  | All patients | | | |  | Drug-only | | | |  | Plus-rTMS | | | |  |
| --- | --- | --- | --- | --- | --- | --- | --- | --- | --- | --- | --- | --- | --- | --- | --- |
| 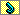 | N | Mean | Median | SD |  | N | Mean | Median | SD |  | N | Mean | Median | SD |  |
| **HAM-D17** |  |  |  |  |  |  |  |  |  |  |  |  |  |  |  |
| Baseline | 929 | 23.64 | 23.00 | 4.58 |  | 530 | 23.94 | 23.00 | 4.97 |  | 399 | 23.24 | 23.00 | 3.97 |  |
| Week 2 | 929 | 12.11 | 11.00 | 5.62 |  | 530 | 13.27 | 12.00 | 6.08 |  | 399 | 10.57 | 10.00 | 4.52 |  |
| Week 4 | 927 | 8.32 | 7.00 | 4.74 |  | 530 | 9.38 | 8.00 | 5.15 |  | 397 | 6.90 | 6.00 | 3.68 |  |
| Week 6 | 922 | 6.63 | 5.00 | 4.50 |  | 528 | 7.39 | 6.00 | 4.80 |  | 394 | 5.61 | 5.00 | 3.82 |  |
| Week 8 | 876 | 6.12 | 5.00 | 4.58 |  | 510 | 6.52 | 6.00 | 4.58 |  | 366 | 5.56 | 4.00 | 4.52 |  |
| **Log-HAM-D17** |  |  |  |  |  |  |  |  |  |  |  |  |  |  |  |
| Baseline | 929 | 3.15 | 3.14 | 0.19 |  | 530 | 3.16 | 3.14 | 0.20 |  | 399 | 3.13 | 3.14 | 0.17 |  |
| Week 2 | 929 | 2.38 | 2.40 | 0.51 |  | 530 | 2.47 | 2.48 | 0.49 |  | 399 | 2.25 | 2.30 | 0.49 |  |
| Week 4 | 927 | 1.96 | 1.95 | 0.59 |  | 530 | 2.09 | 2.08 | 0.57 |  | 397 | 1.79 | 1.79 | 0.56 |  |
| Week 6 | 922 | 1.66 | 1.61 | 0.70 |  | 528 | 1.78 | 1.79 | 0.69 |  | 394 | 1.50 | 1.61 | 0.69 |  |
| Week 8 | 876 | 1.54 | 1.61 | 0.77 |  | 510 | 1.62 | 1.79 | 0.75 |  | 366 | 1.43 | 1.39 | 0.78 |  |

**Table S2.** Factors associated with treatment response measured by HAM-D17 (log-transformed)

|  | Effect | Num DF | Den DF | F Value | P | Sig |
| --- | --- | --- | --- | --- | --- | --- |
|  | Sex | 1 | 3608 | 0.03 | 0.8605 |  |
|  | Age of onset | 1 | 3608 | 4.47 | 0.0346 | * |
|  | Number of episodes | 2 | 3608 | 0.35 | 0.7040 |  |
|  | Duration of illness (month) | 1 | 3608 | 1.28 | 0.2585 |  |
|  | Level of education | 2 | 3608 | 3.06 | 0.0472 | * |
|  | Family history | 1 | 3608 | 0.03 | 0.8521 |  |
|  | Type of drug | 2 | 3608 | 1.22 | 0.2956 |  |
|  |  |  |  |  |  |  |
|  | Treatment | 4 | 3608 | 899.95 | <.0001 | ** |
|  | Treatment * episode | 8 | 3608 | 1.5 | 0.1505 |  |
|  | MDS1 | 1 | 3608 | 2.36 | 0.1243 |  |
|  | rTMS vs. drug | 1 | 3608 | 3.49 | 0.0618 |  |
|  | Treatment by rTMS | 4 | 3608 | 13.69 | <.0001 | ** |

Note: NumDF, numerator degree of freedom; DenDF, denominator degree of freedom.

*, higher education tend to be negatively associated with symptom; and late age of onset is positively associated with symptoms; **, very significant.

**Table S3.** Distribution of rare SNVs by gene regions

|  | | ALL (107,875 SNVs) | | |  |  | MDD* (56,552SNVs ) | | | |
| --- | --- | --- | --- | --- | --- | --- | --- | --- | --- | --- |
| Gene region | | N | % |  |  |  |  | N | % |  |
| UTR3 | | 24,435 | 22.65 |  |  |  |  | 13,236 | 23.41 |  |
| UTR5 | | 5567 | 5.16 |  |  |  |  | 3,001 | 5.31 |  |
| Downstream | | 1308 | 1.21 |  |  |  |  | 717 | 1.27 |  |
| Exonic | | 26,127 | 24.22 |  |  |  |  | 13,663 | 24.16 |  |
| Intergen | | 419 | 0.39 |  |  |  |  | 205 | 0.36 |  |
| Intronic | | 40,179 | 37.25 |  |  |  |  | 20,334 | 35.96 |  |
| ncRNA_exonic | | 640 | 0.59 |  |  |  |  | 324 | 0.57 |  |
| ncRNA_intronic | | 2,300 | 2.13 |  |  |  |  | 1,161 | 2.05 |  |
| ncRNA_splicing | | 15 | 0.01 |  |  |  |  | 8 | 0.01 |  |
| Splicing | | 5,356 | 4.97 |  |  |  |  | 3087 | 5.46 |  |
| Upstream | | 1,529 | 1.42 |  |  |  |  | 816 | 1.44 |  |
| **Function class of exonic variant** |  | |  |  |  | |  |  |  |  |
| Frameshift deletion | 180 | | 0.69 |  |  | |  | 109 | 0.8 |  |
| Frameshift insertion | 2,915 | | 11.16 |  |  | |  | 2,877 | 21.06 |  |
| Nonframeshift deletion | 189 | | 0.72 |  |  | |  | 88 | 0.64 |  |
| Nonframeshift insertion | 65 | | 0.25 |  |  | |  | 34 | 0.25 |  |
| Nonsynonymous | 13,482 | | 51.60 |  |  | |  | 6,519 | 47.71 |  |
| Stop gain | 260 | | 1 |  |  | |  | 152 | 1.11 |  |
| Stop loss | 13 | | 0.05 |  |  | |  | 9 | 0.07 |  |
| Synonymous | 8,846 | | 33.86 |  |  | |  | 3,788 | 27.72 |  |
| Unknown | 177 | | 0.68 |  |  | |  | 87 | 0.64 |  |

MDD*, SNVs detected in MDD but not in controls, CHBS1KG or all samples of 1000 Genomes.

**Tables S4**. Frequency of SNVs consistently called by GATK and Varscan programs by gene regions and functional class

|  |  | ALL | |  |  | MDD* | |
| --- | --- | --- | --- | --- | --- | --- | --- |
|  |  | Freq | % |  |  | Freq | % |
| **Gene region** | |  |  |  |  |  |  |
|  | Downstream | 381 | 0.66 |  |  | 193 | 0.71 |
|  | Exonic | 20,992 | 36.20 |  |  | 9,720 | 35.61 |
|  | Intergen | 195 | 0.34 |  |  | 93 | 0.34 |
|  | Intronic | 10,530 | 18.21 |  |  | 4,862 | 17.86 |
|  | ncRNA_ex | 360 | 0.62 |  |  | 172 | 0.63 |
|  | ncRNA_in | 974 | 1.68 |  |  | 470 | 1.73 |
|  | ncRNA_sp | 6 | 0.01 |  |  | 3 | 0.01 |
|  | Splicing | 3,438 | 5.95 |  |  | 1,673 | 6.15 |
|  | Upstream | 435 | 0.75 |  |  | 198 | 0.73 |
|  | UTR3 | 16,764 | 28.99 |  |  | 8,003 | 29.4 |
|  | UTR5 | 3,755 | 6.47 |  |  | 1833 | 6.72 |
| **Functional variant class** | |  |  |  |  |  |  |
|  | Frameshift deletion | 156 | 0.74 |  |  | 96 | 0.99 |
|  | Frameshift insertion | 278 | 1.32 |  |  | 255 | 2.62 |
|  | Nonframeshift deletion | 139 | 0.66 |  |  | 63 | 0.65 |
|  | Nonframeshift insertion | 41 | 0.2 |  |  | 20 | 0.21 |
|  | Nonsynonymous SNV | 11,988 | 57.11 |  |  | 5,721 | 58.86 |
|  | Stop gain | 212 | 1.01 |  |  | 114 | 1.17 |
|  | Stopl oss | 11 | 0.05 |  |  | 8 | 0.08 |
|  | Synonymous SNV | 8,016 | 38.19 |  |  | 3,375 | 34.72 |
|  | Unknown | 151 | 0.72 |  |  | 68 | 0.7 |

MDD*, SNVs were detected in MDD only, but not in controls or 1000 Genomes

**Table S5.** Summary statistics of mutational load of total107,875 SNVs by gene region and treatment group.

|  |  | All patients (N=929) | | | | |  | Drug-only group (N=530) | | | | |  | Plus-rTMS group (N=399) | | | |  |
| --- | --- | --- | --- | --- | --- | --- | --- | --- | --- | --- | --- | --- | --- | --- | --- | --- | --- | --- |
|  |  | Mean | Med | SD | 90th | Sum |  | Mean | Med | SD | 90th | Sum |  | Mean | Med | SD | 90th | Sum |
| Overall |  | 211.4 | 160 | 248.1 | 193 | 196376 |  | 251.69 | 162 | 322.17 | 224 | 133395 |  | 157.85 | 158 | 22.86 | 185 | 62981 |
| Downstream |  | 2.57 | 2 | 3.56 | 4 | 2392 |  | 3.12 | 2 | 4.46 | 6 | 1653 |  | 1.85 | 2 | 1.44 | 4 | 739 |
| Exonic |  | 47.21 | 35 | 59.12 | 46 | 43858 |  | 56.68 | 35 | 76.76 | 57 | 30042 |  | 34.63 | 34 | 6.35 | 43 | 13816 |
| Intergenic |  | 0.74 | 0 | 1.08 | 2 | 690 |  | 0.84 | 0 | 1.22 | 2 | 446 |  | 0.61 | 0 | 0.85 | 2 | 244 |
| Intronic |  | 82.32 | 64 | 91.84 | 84 | 76476 |  | 97.67 | 65 | 118.80 | 95 | 51764 |  | 61.93 | 62 | 13.38 | 79 | 24712 |
| ncRNA_exonic |  | 1.35 | 1 | 1.65 | 3 | 1251 |  | 1.54 | 1 | 1.93 | 3 | 817 |  | 1.09 | 1 | 1.13 | 3 | 434 |
| ncRNA_intronic |  | 4.47 | 3 | 5.92 | 7 | 4156 |  | 5.40 | 3 | 7.53 | 8 | 2863 |  | 3.24 | 3 | 1.91 | 6 | 1293 |
| ncRNA_splicing |  | 0.04 | 0 | 0.21 | 0 | 39 |  | 0.03 | 0 | 0.18 | 0 | 17 |  | 0.06 | 0 | 0.24 | 0 | 22 |
| Splicing |  | 11.62 | 8 | 18.54 | 13 | 10795 |  | 14.28 | 8 | 24.07 | 16 | 7569 |  | 8.09 | 8 | 3.13 | 12 | 3226 |
| Upstream |  | 3.00 | 2 | 3.89 | 5 | 2790 |  | 3.57 | 2 | 4.91 | 6 | 1893 |  | 2.25 | 2 | 1.51 | 4 | 897 |
| UTR3 |  | 48.31 | 37 | 55.97 | 50 | 44879 |  | 57.00 | 37 | 72.65 | 57.5 | 30211 |  | 36.76 | 37 | 7.45 | 46 | 14668 |
| UTR5 |  | 10.41 | 8 | 11.95 | 13 | 9669 |  | 12.42 | 8 | 15.30 | 16 | 6585 |  | 7.73 | 8 | 3.03 | 12 | 3084 |
| Frameshift_deletion |  | 0.19 | 0 | 0.45 | 1 | 177 |  | 0.18 | 0 | 0.43 | 1 | 98 |  | 0.20 | 0 | 0.49 | 1 | 79 |
| Frameshift_insertion |  | 12.46 | 0 | 57.95 | 1 | 11574 |  | 21.76 | 0 | 75.43 | 2 | 11533 |  | 0.10 | 0 | 0.32 | 0 | 41 |
| Nonframeshift_deleletion |  | 0.31 | 0 | 0.56 | 1 | 284 |  | 0.30 | 0 | 0.53 | 1 | 160 |  | 0.31 | 0 | 0.60 | 1 | 124 |
| Nonframeshift_insertion |  | 0.11 | 0 | 0.35 | 1 | 106 |  | 0.11 | 0 | 0.35 | 0 | 57 |  | 0.12 | 0 | 0.34 | 1 | 49 |
| Nonsynonymous |  | 19.12 | 19 | 4.63 | 25 | 17765 |  | 19.34 | 19 | 4.80 | 26 | 10250 |  | 18.83 | 18 | 4.37 | 25 | 7515 |
| Stop gain |  | 0.27 | 0 | 0.53 | 1 | 247 |  | 0.27 | 0 | 0.56 | 1 | 145 |  | 0.26 | 0 | 0.50 | 1 | 102 |
| Synonymous |  | 14.43 | 14 | 3.98 | 20 | 13408 |  | 14.37 | 14 | 3.99 | 20 | 7615 |  | 14.52 | 14 | 3.97 | 20 | 5793 |
| Unknown |  | 0.29 | 0 | 0.64 | 1 | 274 |  | 0.31 | 0 | 0.71 | 1 | 165 |  | 0.27 | 0 | 0.52 | 1 | 109 |

**Table S6.** Mutational load of total 107,875 SNVs impact antidepressant response by gene region and treatment group

|  |  | All patients (N=929) | |  |  | Drug-only group(N=530) | | |  | Plus-rTMS group (N=399) | | |
| --- | --- | --- | --- | --- | --- | --- | --- | --- | --- | --- | --- | --- |
| Region or function | DenDF | Pnonlog | P_log | Pmin | denDF | P_nonlog | P_log | Pmin | denDF | P_nonlog | P_log | Pmin |
| Overall | 3605 | 2.84E-04 | 3.61E-05 | 3.61E-05 | 2052 | 2.03E-04 | 5.13E-05 | 5.13E-05 | 1539 | 1.25E-01 | 1.42E-01 | 1.25E-01 |
| Downstream | 3604 | 3.96E-04 | 1.42E-03 | 3.96E-04 | 2052 | 5.02E-04 | 9.11E-04 | 5.02E-04 | 1539 | 5.15E-01 | 2.07E-01 | 2.07E-01 |
| Exonic variants | 3604 | 7.32E-04 | 7.43E-04 | 7.32E-04 | 2052 | 3.23E-04 | 2.44E-04 | 2.44E-04 | 1539 | 1.62E-01 | 1.32E-01 | 1.32E-01 |
| Intergenic | 3606 | 4.09E-03 | 4.62E-03 | 4.09E-03 | 2052 | 3.73E-02 | 4.65E-02 | 3.73E-02 | 1540 | 1.32E-02 | 2.37E-03 | 2.37E-03 |
| Intronic | 3606 | 1.22E-04 | 1.72E-06 | 1.72E-06 | 2052 | 1.04E-04 | 4.41E-06 | 4.41E-06 | 1539 | 7.07E-02 | 4.28E-02 | 4.28E-02 |
| ncRNA_exonic | 3606 | 8.93E-04 | 1.29E-03 | 8.93E-04 | 2052 | 5.82E-03 | 5.31E-03 | 5.31E-03 | 1539 | 1.29E-02 | 1.74E-02 | 1.29E-02 |
| ncRNA_intronic | 3606 | 1.43E-03 | 6.56E-02 | 1.43E-03 | 2052 | 3.76E-04 | 2.80E-02 | 3.76E-04 | 1539 | 2.00E-02 | 4.21E-02 | 2.00E-02 |
| ncRNA_splicing | 3605 | 5.64E-01 | 9.61E-01 | 5.64E-01 | 2052 | 2.37E-01 | NA | 2.37E-01 | 1540 | 3.21E-01 | 9.72E-01 | 3.21E-01 |
| Splicing | 3605 | 5.00E-04 | 5.77E-04 | 5.00E-04 | 2052 | 5.32E-04 | 2.46E-03 | 5.32E-04 | 1539 | 1.50E-01 | 1.23E-01 | 1.23E-01 |
| Upstream |  | 3.48E-04 | 1.63E-03 | 3.48E-04 | 2052 | 1.74E-04 | 1.33E-04 | 1.33E-04 | 1539 | 4.13E-01 | 5.54E-01 | 4.13E-01 |
| UTR3 | 3603 | 4.24E-04 | 7.96E-04 | 4.24E-04 | 2052 | 3.39E-04 | 1.24E-03 | 3.39E-04 | 1540 | 2.71E-01 | 2.79E-01 | 2.71E-01 |
| UTR5 | 3605 | 1.46E-03 | 5.75E-03 | 1.46E-03 | 2052 | 6.67E-04 | 2.94E-03 | 6.67E-04 | 1539 | 9.71E-02 | 1.73E-01 | 9.71E-02 |
| Frameshift_deletion | 3605 | 1.62E-01 | 1.83E-01 | 1.62E-01 | 2053 | 1.49E-01 | 2.90E-01 | 1.49E-01 | 1540 | 5.07E-01 | 5.10E-01 | 5.07E-01 |
| Frameshift_insertion | 3603 | 1.08E-03 | 1.83E-01 | 1.08E-03 | 2052 | 5.77E-04 | 8.22E-04 | 5.77E-04 | 1539 | 8.14E-01 | 9.50E-01 | 8.14E-01 |
| Nonframeshift_deletion | 3606 | 4.47E-03 | 4.39E-02 | 4.47E-03 | 2052 | 1.13E-01 | 1.50E-01 | 1.13E-01 | 1539 | 2.86E-02 | 8.00E-02 | 2.86E-02 |
| Nonframeshift_ins | 3605 | 4.35E-01 | 4.09E-01 | 4.09E-01 | 2052 | 1.61E-01 | 2.32E-01 | 1.61E-01 | 1539 | 8.41E-01 | 9.69E-01 | 8.41E-01 |
| Nonsynonymous | 3606 | 6.09E-01 | 5.32E-01 | 5.32E-01 | 2052 | 4.25E-01 | 4.92E-01 | 4.25E-01 | 1539 | 7.89E-01 | 7.02E-01 | 7.02E-01 |
| Stop gain | 3604 | 2.75E-01 | 7.42E-01 | 2.75E-01 | 2052 | 1.03E-01 | 2.88E-01 | 1.03E-01 | 1539 | 3.42E-01 | 5.26E-01 | 3.42E-01 |
| Synonymous | 3605 | 1.93E-02 | 9.17E-03 | 9.17E-03 | 2051 | 5.50E-02 | 5.59E-02 | 5.50E-02 | 1539 | 3.50E-01 | 1.74E-01 | 1.74E-01 |
| Unknown | 3605 | 7.30E-02 | 8.95E-02 | 7.30E-02 | 2053 | 8.14E-03 | 1.04E-02 | 8.14E-03 | 1540 | 6.07E-01 | 9.74E-01 | 6.07E-01 |

Note: SNVs detected only in patients with MDD but not in 1000 Genomes or controls; P_log, p value for log-trasnformed SNV burden; P_nonlog, p value for non-log transformed; P_min, minumum p value of two.

**Table S7.** Summary statistics of burden of 56,552 SNVs* detected in MDD only by gene region and treatment group

|  |  | All patients (N=929) | | | | |  | Drug-only group(N=530) | | | | |  | Plus-rTMS group(N=399) | | | | |  |
| --- | --- | --- | --- | --- | --- | --- | --- | --- | --- | --- | --- | --- | --- | --- | --- | --- | --- | --- | --- |
| Region or function | N | Mean | Median | SD | 90th | Sum | N | Mean | Median | SD | 90th | Sum | N | Mean | Median | SD | 90th | Sum |  |
| Overall | 929 | 103.83 | 53.00 | 240.93 | **67.00** | 96455 | 530 | 143.52 | 54.00 | 313.19 | 79 | 76067 | 399 | 51.10 | 51.00 | 9.00 | 62 | 20388 |  |
| Downstream | 929 | 1.288 | 0 | 3.16 | 2 | 1197 | 530 | 1.79 | 1.00 | 4.05 | 3 | 949 | 399 | 0.62 | 0 | 0.79 | 2 | 248 |  |
| Exonic | 929 | 24.693 | 12 | 58.35 | 18 | 22940 | 530 | 34.21 | 13.00 | 75.84 | 24 | 18132 | 399 | 12.05 | 12 | 3.58 | 17 | 4808 |  |
| Intergenic | 929 | 0.313 | 0 | 0.75 | 1 | 291 | 530 | 0.41 | 0.00 | 0.90 | 1 | 215 | 399 | 0.19 | 0 | 0.47 | 1 | 76 |  |
| Intronic | 929 | 37.823 | 19 | 88.43 | 28 | 35138 | 530 | 52.75 | 20.00 | 114.80 | 36 | 27957 | 399 | 18.00 | 18 | 5.17 | 25 | 7181 |  |
| ncRNA_exonic | 929 | 0.549 | 0 | 1.23 | 1 | 510 | 530 | 0.72 | 0.00 | 1.53 | 2 | 381 | 399 | 0.32 | 0 | 0.58 | 1 | 129 |  |
| ncRNA_intronic | 929 | 2.191 | 1 | 5.40 | 3 | 2035 | 530 | 3.07 | 1.00 | 6.96 | 4 | 1627 | 399 | 1.02 | 1 | 1.03 | 2 | 408 |  |
| ncRNA_splicing | 929 | 0.024 | 0 | 0.16 | 0 | 22 | 530 | 0.02 | 0.00 | 0.13 | 0 | 9 | 399 | 0.03 | 0 | 0.19 | 0 | 13 |  |
| Splicing | 929 | 6.489 | 3 | 17.86 | 6 | 6028 | 530 | 9.24 | 3.00 | 23.23 | 7 | 4897 | 399 | 2.83 | 3 | 1.83 | 5 | 1131 |  |
| Upstream | 929 | 1.519 | 1 | 3.66 | 2 | 1411 | 530 | 2.10 | 1.00 | 4.70 | 3.5 | 1112 | 399 | 0.75 | 1 | 0.84 | 2 | 299 |  |
| UTR3 | 929 | 23.761 | 13 | 52.95 | 19 | 22074 | 530 | 32.22 | 13.00 | 68.86 | 23 | 17075 | 399 | 12.53 | 12 | 3.78 | 18 | 4999 |  |
| UTR5 | 929 | 5.177 | 3 | 11.22 | 6 | 4809 | 530 | 7.01 | 3.00 | 14.52 | 8.5 | 3713 | 399 | 2.75 | 2 | 1.76 | 5 | 1096 |  |
| Frameshift_deletion | 929 | 0.131 | 0 | 0.37 | 1 | 122 | 530 | 0.13 | 0.00 | 0.35 | 1 | 68 | 399 | 0.14 | 0 | 0.40 | 1 | 54 |  |
| Frameshift_insertion | 929 | 12.352 | 0 | 57.67 | 1 | 11475 | 530 | 21.61 | 0.00 | 75.06 | 2 | 11452 | 399 | 0.06 | 0 | 0.24 | 0 | 23 |  |
| Nnonframeshift_deletion | 929 | 0.099 | 0 | 0.32 | 0 | 92 | 530 | 0.10 | 0.00 | 0.32 | 0 | 52 | 399 | 0.10 | 0 | 0.32 | 0 | 40 |  |
| Nnonframeshift_insertion | 929 | 0.044 | 0 | 0.22 | 0 | 41 | 530 | 0.04 | 0.00 | 0.21 | 0 | 21 | 399 | 0.05 | 0 | 0.22 | 0 | 20 |  |
| Nonsynonymous | 929 | 7.354 | 7 | 2.77 | 11 | 6832 | 530 | 7.52 | 7.00 | 2.76 | 11 | 3983 | 399 | 7.14 | 7 | 2.77 | 11 | 2849 |  |
| Stop gain | 929 | 0.171 | 0 | 0.44 | 1 | 159 | 530 | 0.17 | 0.00 | 0.46 | 1 | 91 | 399 | 0.17 | 0 | 0.41 | 1 | 68 |  |
| Synonymous | 929 | 4.352 | 4 | 2.03 | 7 | 4043 | 530 | 4.42 | 4.00 | 1.99 | 7 | 2342 | 399 | 4.26 | 4 | 2.08 | 7 | 1701 |  |
| Unknown | 929 | 0.173 | 0 | 0.53 | 1 | 161 | 530 | 0.21 | 0.00 | 0.63 | 1 | 110 | 399 | 0.13 | 0 | 0.35 | 1 | 51 |  |

***,** SNVs detected in only patients with MDD but not in 1000 Genomes or controls

**Table S8.** Burden of 56,552 SNVs detected in MDD only on antidepressant response by gene region and treatment group

|  |  | All patients (N=929) | |  |  | Drug-only (n=530) | |  |  | Plus-rTMS (n=399) | |
| --- | --- | --- | --- | --- | --- | --- | --- | --- | --- | --- | --- |
| Region or function | P_nonlog | P_log | P_min |  | P_nonlog | P_log | P_min |  | P_nonlog | P_log | P_min |
| Overall | 6.11E-04 | 7.82E-05 | 7.82E-05 |  | 3.38E-04 | 6.13E-05 | 6.13E-05 |  | 7.83E-01 | 7.83E-01 | 7.83E-01 |
| Downstream | 7.86E-04 | 1.85E-03 | 7.86E-04 |  | 6.94E-04 | 1.04E-03 | 6.94E-04 |  | 1.72E-02 | 2.92E-02 | 1.72E-02 |
| Exonic | 8.61E-04 | 4.57E-04 | 4.57E-04 |  | 4.82E-04 | 7.22E-04 | 4.82E-04 |  | 1.07E-01 | 2.74E-02 | 2.74E-02 |
| Intergen | 4.72E-04 | 4.80E-03 | 4.72E-04 |  | 8.73E-04 | 1.66E-03 | 8.73E-04 |  | 3.13E-01 | 8.79E-01 | 3.13E-01 |
| Intronic | 5.29E-04 | 4.79E-05 | 4.79E-05 |  | 2.81E-04 | 1.30E-05 | 1.30E-05 |  | 6.68E-01 | 6.91E-01 | 6.68E-01 |
| ncRNA_ex | 1.80E-03 | 9.02E-04 | 9.02E-04 |  | 1.67E-04 | 1.03E-04 | 1.03E-04 |  | 2.72E-01 | 7.50E-01 | 2.72E-01 |
| ncRNA_intronic | 2.60E-03 | 1.42E-01 | 2.60E-03 |  | 9.00E-04 | 1.79E-02 | 9.00E-04 |  | 2.69E-01 | 3.72E-01 | 2.69E-01 |
| ncRNA_splicing | 5.55E-01 | 9.61E-01 | 5.55E-01 |  | 1.72E-01 | NA | 1.72E-01 |  | 4.04E-01 | 9.72E-01 | 4.04E-01 |
| Splicing | 5.25E-04 | 5.80E-04 | 5.25E-04 |  | 3.91E-04 | 4.01E-04 | 3.91E-04 |  | 2.04E-01 | 1.26E-01 | 1.26E-01 |
| Upstream | 5.54E-04 | 1.62E-04 | 1.62E-04 |  | 5.89E-04 | 2.45E-04 | 2.45E-04 |  | 4.16E-02 | 3.18E-02 | 3.18E-02 |
| UTR3 | 5.82E-04 | 9.48E-04 | 5.82E-04 |  | 3.06E-04 | 3.53E-04 | 3.06E-04 |  | 7.89E-01 | 8.29E-01 | 7.89E-01 |
| UTR5 | 2.60E-03 | 2.97E-02 | 2.60E-03 |  | 1.24E-03 | 8.72E-03 | 1.24E-03 |  | 5.15E-02 | 1.81E-01 | 5.15E-02 |
| Frameshift_deletion | 2.87E-01 | 5.15E-01 | 2.87E-01 |  | 2.71E-01 | 5.09E-01 | 2.71E-01 |  | 6.79E-01 | 7.56E-01 | 6.79E-01 |
| Frameshift_insertion | 1.10E-03 | 1.34E-03 | 1.10E-03 |  | 5.79E-04 | 7.77E-04 | 5.79E-04 |  | 8.61E-01 | 9.41E-01 | 8.61E-01 |
| Nonframeshift_deletion | 2.34E-01 | 2.03E-01 | 2.03E-01 |  | 4.70E-01 | 9.01E-01 | 4.70E-01 |  | 6.65E-01 | 4.13E-02 | 4.13E-02 |
| Nonframeshift_insertion | 7.08E-01 | 7.19E-01 | 7.08E-01 |  | 3.22E-01 | 6.68E-01 | 3.22E-01 |  | 5.43E-01 | . | 5.43E-01 |
| Nonsynonymous | 3.68E-01 | 2.73E-01 | 2.73E-01 |  | 7.01E-01 | 7.31E-01 | 7.01E-01 |  | 4.19E-01 | 3.28E-01 | 3.28E-01 |
| Stop gain | 1.57E-01 | 4.37E-01 | 1.57E-01 |  | 5.61E-01 | 5.71E-01 | 5.61E-01 |  | 1.43E-02 | 5.14E-01 | 1.43E-02 |
| Synonymous | 2.02E-01 | 1.55E-01 | 1.55E-01 |  | 5.55E-01 | 9.53E-01 | 5.55E-01 |  | 5.39E-01 | 1.04E-01 | 1.04E-01 |
| Unknown | 9.80E-03 | 1.04E-02 | 9.80E-03 |  | 7.00E-04 | 5.38E-03 | 7.00E-04 |  | 8.57E-01 | 6.77E-01 | 6.77E-01 |

Note: SNVs detected only in patients with MDD but not in 1000 Genomes or controls; P_log, p value for log-trasnformed SNV burden; P_nonlog, p value for non-log transformed; P_min, minumum p value of two.

**Table S9**. Higer level of burden SNVs (the 90th percentile, burden>67) on antidepressant response in overall patient and by treatment group

|  |  | Least-square mean | |  |  |  |  |  |  |
| --- | --- | --- | --- | --- | --- | --- | --- | --- | --- |
|  | 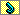TIME | Burden<=90% | Burden>90% | Estimate | SE | DF | t Value | P | P_interaction |
| Overall |  |  |  |  |  |  |  |  |  |
|  | Baseline | 3.1725 | 3.0666 | 0.1059 | 0.0643 | 3608 | 1.6500 | 0.0999 | 3.90E-05 |
|  | 2 week | 2.4548 | 2.6116 | -0.1568 | 0.0643 | 3608 | -2.4400 | 0.0149 |  |
|  | 4 week | 2.0072 | 2.1807 | -0.1735 | 0.0643 | 3608 | -2.7000 | 0.0070 |  |
|  | 6 week | 1.7252 | 1.8482 | -0.1230 | 0.0644 | 3608 | -1.9100 | 0.0561 |  |
|  | 8 week | 1.6021 | 1.6188 | -0.0168 | 0.0653 | 3608 | -0.2600 | 0.7973 |  |
| Drug-only |  |  |  |  |  |  |  |  |  |
|  | Baseline | 3.1317 | 3.0193 | 0.1124 | 0.0715 | 2052 | 1.5700 | 0.1159 | 1.23E-04 |
|  | 2 week | 2.4684 | 2.6337 | -0.1653 | 0.0715 | 2052 | -2.3100 | 0.0209 |  |
|  | 4 week | 2.0740 | 2.2435 | -0.1695 | 0.0715 | 2052 | -2.3700 | 0.0178 |  |
|  | 6 week | 1.7912 | 1.9256 | -0.1345 | 0.0715 | 2052 | -1.8800 | 0.0602 |  |
|  | 8 week | 1.6336 | 1.6542 | -0.0206 | 0.0724 | 2052 | -0.2800 | 0.7757 |  |
| Plus-rTMS |  |  |  |  |  |  |  |  |  |
|  | Baseline | 3.1465 | 3.2513 | -0.1048 | 0.1454 | 1539 | -0.7200 | 0.4710 | 0.7926 |
|  | 2 week | 2.3410 | 2.4328 | -0.0919 | 0.1454 | 1539 | -0.6300 | 0.5275 |  |
|  | 4 week | 1.7758 | 1.8193 | -0.0435 | 0.1454 | 1539 | -0.3000 | 0.7648 |  |
|  | 6 week | 1.4891 | 1.4386 | 0.0505 | 0.1454 | 1539 | 0.3500 | 0.7284 |  |
|  | 8 week | 1.4232 | 1.3864 | 0.0658 | 0.0598 | 1539 | 1.1000 | 0.2712 |  |

**Table S10.** Summary statistics of the burden of all 57,830 SNVs consistently called by GATK and Varscan programs by gene region and treatment group.

|  |  | All patients (N=929) | | | | |  | Drug-only group (N=530) | | | | |  | Plus-rTMS group (N=399) | | | | |
| --- | --- | --- | --- | --- | --- | --- | --- | --- | --- | --- | --- | --- | --- | --- | --- | --- | --- | --- |
| Region or function | N | Mean | Median | SD | 90th | Sum | N | Mean | Median | SD | 90th | Sum | N | Mean | Median | SD | 90th | Sum |
| Overall | 929 | 87.614 | 87 | 15.954 | 104 | 81393 | 530 | 88.82 | 87.00 | 17.71 | 107.5 | 47077 | 399 | 86.01 | 86.00 | 13.12 | 102 | 34316 |
| Downstream | 929 | 0.539 | 0 | 0.762 | 2 | 501 | 530 | 0.56 | 0.00 | 0.79 | 2 | 299 | 399 | 0.51 | 0.00 | 0.73 | 1 | 202 |
| Exonic | 929 | 30.569 | 30 | 6.437 | 39 | 28399 | 530 | 30.83 | 30.00 | 6.62 | 39 | 16341 | 399 | 30.22 | 30.00 | 6.18 | 38 | 12058 |
| Intergenic | 929 | 0.292 | 0 | 0.559 | 1 | 271 | 530 | 0.28 | 0.00 | 0.57 | 1 | 150 | 399 | 0.30 | 0.00 | 0.54 | 1 | 121 |
| Intronic | 929 | 16.709 | 16 | 5.386 | 23 | 15523 | 530 | 17.11 | 16.00 | 5.86 | 24 | 9067 | 399 | 16.18 | 16.00 | 4.63 | 22 | 6456 |
| ncRNA_exonic | 929 | 0.598 | 0 | 0.878 | 2 | 556 | 530 | 0.65 | 0.00 | 0.94 | 2 | 345 | 399 | 0.53 | 0.00 | 0.79 | 2 | 211 |
| ncRNA_intronic | 929 | 1.489 | 1 | 1.273 | 3 | 1383 | 530 | 1.56 | 1.00 | 1.30 | 3 | 826 | 399 | 1.40 | 1.00 | 1.23 | 3 | 557 |
| Splicing | 929 | 5.110 | 5 | 2.498 | 8 | 4747 | 530 | 5.16 | 5.00 | 2.67 | 8 | 2733 | 399 | 5.05 | 5.00 | 2.26 | 8 | 2014 |
| Upstream | 929 | 0.622 | 0 | 0.811 | 2 | 578 | 530 | 0.61 | 0.00 | 0.81 | 2 | 325 | 399 | 0.63 | 0.00 | 0.82 | 2 | 253 |
| UTR3 | 929 | 26.191 | 26 | 6.802 | 35 | 24331 | 530 | 26.40 | 26.00 | 7.31 | 36 | 13994 | 399 | 25.91 | 26.00 | 6.05 | 33 | 10337 |
| UTR5 | 929 | 5.471 | 5 | 2.485 | 9 | 5083 | 530 | 5.64 | 5.00 | 2.53 | 9 | 2987 | 399 | 5.25 | 5.00 | 2.40 | 8 | 2096 |
| Frameshift_deletion | 929 | 0.157 | 0 | 0.409 | 1 | 146 | 530 | 0.16 | 0.00 | 0.40 | 1 | 83 | 399 | 0.16 | 0.00 | 0.42 | 1 | 63 |
| Frameshift_insertion | 929 | 0.445 | 0 | 2.055 | 1 | 413 | 530 | 0.72 | 0.00 | 2.68 | 1 | 384 | 399 | 0.07 | 0.00 | 0.26 | 0 | 29 |
| Nonframeshift_del | 929 | 0.220 | 0 | 0.466 | 1 | 204 | 530 | 0.21 | 0.00 | 0.44 | 1 | 110 | 399 | 0.24 | 0.00 | 0.50 | 1 | 94 |
| Nonframeshift_ins | 929 | 0.040 | 0 | 0.201 | 0 | 37 | 530 | 0.03 | 0.00 | 0.18 | 0 | 15 | 399 | 0.06 | 0.00 | 0.23 | 0 | 22 |
| Nonsynonymous | 929 | 16.487 | 16 | 4.325 | 22 | 15316 | 530 | 16.65 | 16.00 | 4.45 | 22 | 8822 | 399 | 16.28 | 16.00 | 4.14 | 22 | 6494 |
| Stop gain | 929 | 0.206 | 0 | 0.459 | 1 | 191 | 530 | 0.19 | 0.00 | 0.46 | 1 | 103 | 399 | 0.22 | 0.00 | 0.46 | 1 | 88 |
| Synonymous | 929 | 12.818 | 13 | 3.807 | 18 | 11908 | 530 | 12.70 | 13.00 | 3.79 | 17.5 | 6733 | 399 | 12.97 | 13.00 | 3.84 | 18 | 5175 |
| Unknown | 929 | 0.191 | 0 | 0.437 | 1 | 177 | 530 | 0.16 | 0.00 | 0.41 | 1 | 86 | 399 | 0.23 | 0.00 | 0.47 | 1 | 91 |

**Table S11.** Burden of 57,830 SNVs* consistently called by GATK and Varscan programs affect antidepressant response by gene region and treatment group

|  |  | All patients (N=929) | | |  | Drug-only group(N=530) | | |  | Plus-rTMS group (N=399) | | |
| --- | --- | --- | --- | --- | --- | --- | --- | --- | --- | --- | --- | --- |
| Gene region or function | Den DF | P_nonlog | P_log | P_min | Den DF | P_nonlog | P_log | P_min | Den DF | P_nonlog | P_log | P_min |
| Overall | 3602 | 0.0008 | 0.0032 | 8.0E-04 | 2050 | 0.0003 | 0.0029 | 0.0003 | 1539 | 0.6032 | 0.5522 | 0.5522 |
| Frameshift Insertion | 3602 | 0.0057 | 0.0013 | 1.3E-03 | 2050 | 0.0035 | 0.0007 | 0.0007 | 1539 | 0.9925 | . | 0.9925 |
| Intronic | 3600 | 0.0087 | 0.0203 | 8.7E-03 | 2049 | 0.0129 | 0.0169 | 0.0129 | 1540 | 0.3077 | 0.483 | 0.3077 |
| Intergenic | 3600 | 0.0126 | 0.1437 | 1.3E-02 | 2049 | 0.7359 | 0.7361 | 0.7359 | 1540 | 0.0020 | 0.1135 | 0.0020 |
| Splicing | 3601 | 0.017 | 0.1124 | 1.7E-02 | 2050 | 0.0471 | 0.3917 | 0.0471 | 1539 | 0.1064 | 0.1053 | 0.1053 |
| Exonic | 3601 | 0.0226 | 0.0240 | 2.3E-02 | 2050 | 0.0216 | 0.0479 | 0.0216 | 1539 | 0.0718 | 0.0638 | 0.0638 |
| UTR3 | 3601 | 0.0278 | 0.0919 | 2.8E-02 | 2050 | 0.0205 | 0.0807 | 0.0205 | 1540 | 0.7278 | 0.7363 | 0.7278 |
| Synonymous | 3601 | 0.0893 | 0.0287 | 2.9E-02 | 2050 | 0.1329 | 0.0674 | 0.0674 | 1539 | 0.6402 | 0.4214 | 0.4214 |
| Nonframeshift Insertion | 3602 | 0.0491 | 0.0983 | 4.9E-02 | 2050 | 0.0073 | 0.0721 | 0.0073 | 1539 | 0.1969 | . | 0.1969 |
| Frameshift Deletion | 3602 | 0.0762 | 0.1901 | 0.0762 | 2050 | 0.1568 | 0.128 | 0.1280 | 1539 | 0.2658 | 0.7541 | 0.2658 |
| Nonframeshift deletion | 3602 | 0.1551 | 0.1233 | 0.1233 | 2050 | 0.7071 | 0.2416 | 0.2416 | 1539 | 0.1522 | 0.0378 | 0.0378 |
| Nonsynonymous | 3601 | 0.2931 | 0.1833 | 0.1833 | 2050 | 0.2 | 0.1829 | 0.1829 | 1539 | 0.2496 | 0.2017 | 0.2017 |
| Stop gain | 3602 | 0.3009 | 0.5729 | 0.3009 | 2050 | 0.0668 | 0.0205 | 0.0205 | 1539 | 0.1525 | 0.6537 | 0.1525 |
| ncRNA_exonic | 3601 | 0.8063 | 0.3034 | 0.3034 | 2050 | 0.9482 | 0.322 | 0.3220 | 1539 | 0.6694 | 0.942 | 0.6694 |
| Downstream | 3602 | 0.3424 | 0.6387 | 0.3424 | 2050 | 0.082 | 0.4606 | 0.0820 | 1539 | 0.1634 | 0.8494 | 0.1634 |
| Unknown | 3601 | 0.3805 | 0.9312 | 0.3805 | 2050 | 0.7335 | 0.5335 | 0.5335 | 1540 | 0.4818 | 0.987 | 0.4818 |
| Upstream | 3602 | 0.4046 | 0.8779 | 0.4046 | 2050 | 0.6455 | 0.9447 | 0.6455 | 1540 | 0.6932 | 0.844 | 0.6932 |
| ncRNA_intronic | 3601 | 0.8373 | 0.4375 | 0.4375 | 2049 | 0.6227 | 0.39 | 0.3900 | 1539 | 0.322 | 0.1401 | 0.1401 |
| UTR5 | 3602 | 0.7562 | 0.7759 | 0.7562 | 2050 | 0.2956 | 0.3066 | 0.2956 | 1539 | 0.574 | 0.8238 | 0.5740 |

Note: SNVs consistently called by GATK and Varscan programs**;**

* SNVs detected in patients with MDD but not in 1000 Genomes or controls; P_log, p value for log-trasnformed SNV burden; P_nonlog, p value for non-log transformed; P_min, minumum p value of two.

**Table S12.** Summary statistics of the burden of 27,220 SNVs detected in MDD only and consistently called by GATK and Varscan programs by gene region and treatment group

|  |  | All patients (N=929) | | | | |  | Drug-only group (N=530) | | | | |  | Plus-rTMS group(N=399) | | | | |
| --- | --- | --- | --- | --- | --- | --- | --- | --- | --- | --- | --- | --- | --- | --- | --- | --- | --- | --- |
| Gene region or function | N | Mean | Med | SD | 90th | Sum | N | Mean | Med | SD | 90th | Sum | N | Mean | Med | SD | 90th | Sum |
| Overall | 929 | 31.559 | 30 | 10.4 | 39 | 29318 | 530 | 33.2 | 31 | 12.364 | 41.5 | 17596 | 399 | 29.378 | 30 | 6.37 | 38 | 11722 |
| Downstream | 929 | 0.2271 | 0 | 0.501 | 1 | 211 | 530 | 0.242 | 0 | 0.5309 | 1 | 128 | 399 | 0.208 | 0 | 0.459 | 1 | 83 |
| Exonic | 929 | 11.099 | 11 | 3.764 | 16 | 10311 | 530 | 11.48 | 11 | 3.9583 | 16 | 6085 | 399 | 10.591 | 10 | 3.429 | 15 | 4226 |
| Intergenic | 929 | 0.113 | 0 | 0.361 | 0 | 105 | 530 | 0.117 | 0 | 0.3708 | 1 | 62 | 399 | 0.1078 | 0 | 0.349 | 0 | 43 |
| Intronic | 929 | 5.7589 | 5 | 3.511 | 9 | 5350 | 530 | 6.289 | 6 | 4.1295 | 10 | 3333 | 399 | 5.0551 | 5 | 2.283 | 8 | 2017 |
| ncRNA_exonic | 929 | 0.2088 | 0 | 0.466 | 1 | 194 | 530 | 0.23 | 0 | 0.4879 | 1 | 122 | 399 | 0.1805 | 0 | 0.434 | 1 | 72 |
| ncRNA_intron | 929 | 0.5554 | 0 | 0.757 | 2 | 516 | 530 | 0.628 | 0 | 0.8085 | 2 | 333 | 399 | 0.4586 | 0 | 0.671 | 1 | 183 |
| Splicing | 929 | 1.9473 | 2 | 1.655 | 4 | 1809 | 530 | 2.045 | 2 | 1.8036 | 4 | 1084 | 399 | 1.817 | 2 | 1.425 | 4 | 725 |
| Upstream | 929 | 0.2131 | 0 | 0.471 | 1 | 198 | 530 | 0.23 | 0 | 0.4801 | 1 | 122 | 399 | 0.1905 | 0 | 0.458 | 1 | 76 |
| UTR3 | 929 | 9.3208 | 9 | 3.931 | 14 | 8659 | 530 | 9.728 | 9 | 4.4149 | 15 | 5156 | 399 | 8.7794 | 9 | 3.101 | 13 | 3503 |
| UTR5 | 929 | 2.1087 | 2 | 1.619 | 4 | 1959 | 530 | 2.204 | 2 | 1.7139 | 4 | 1168 | 399 | 1.9825 | 2 | 1.476 | 4 | 791 |
| Frameshift_deletion | 929 | 0.1109 | 0 | 0.34 | 1 | 103 | 530 | 0.113 | 0 | 0.3289 | 1 | 60 | 399 | 0.1078 | 0 | 0.356 | 0 | 43 |
| Frameshift_insertion | 929 | 0.4069 | 0 | 2.044 | 0 | 378 | 530 | 0.683 | 0 | 2.6684 | 1 | 362 | 399 | 0.0401 | 0 | 0.196 | 0 | 16 |
| Nonframeshift_deletion | 929 | 0.0721 | 0 | 0.263 | 0 | 67 | 530 | 0.072 | 0 | 0.2582 | 0 | 38 | 399 | 0.0727 | 0 | 0.269 | 0 | 29 |
| Nonframeshift_insertion | 929 | 0.0269 | 0 | 0.168 | 0 | 25 | 530 | 0.019 | 0 | 0.1494 | 0 | 10 | 399 | 0.0376 | 0 | 0.19 | 0 | 15 |
| Nonsynonymous | 929 | 6.4101 | 6 | 2.614 | 10 | 5955 | 530 | 6.515 | 6 | 2.5583 | 10 | 3453 | 399 | 6.2707 | 6 | 2.684 | 10 | 2502 |
| Stop gain | 929 | 0.1227 | 0 | 0.363 | 1 | 114 | 530 | 0.106 | 0 | 0.3481 | 0 | 56 | 399 | 0.1454 | 0 | 0.38 | 1 | 58 |
| Synonymous | 929 | 3.8644 | 4 | 1.918 | 6 | 3590 | 530 | 3.902 | 4 | 1.8826 | 6 | 2068 | 399 | 3.8145 | 4 | 1.966 | 6 | 1522 |
| Unknown | 929 | 0.0786 | 0 | 0.273 | 0 | 73 | 530 | 0.064 | 0 | 0.2528 | 0 | 34 | 399 | 0.0977 | 0 | 0.297 | 0 | 39 |

Note: SNVs detected only in patients with MDD but not in 1000 Genomes or controls and consistently called by GATK and varscan

**Table S13.** Burden of 27,220 SNVs detected only in MDD and consistently called by GATK and Varscan programs affect antidepressant response by gene region and treatment group

|  |  | All patients (N=929) | | |  | Drug-only group (N=530) | | |  | Plus-rTMS (N=399) | | |
| --- | --- | --- | --- | --- | --- | --- | --- | --- | --- | --- | --- | --- |
| Gene region or function |  | P_nonlog | P_log | P_min | Den DF | P_nonlog | P_log | P_min | Den DF | P_nonlog | P_log | P_min |
| Overall | 3601 | 2.48E-05 | 4.34E-05 | 2.48E-05 | 2050 | 4.34E-05 | 9.03E-05 | 4.34E-05 | 1539 | 0.0621 | 0.0437 | 0.0437 |
| Exonic | 3602 | 5.00E-04 | 2.00E-04 | 2.00E-04 | 2050 | 6.59E-05 | 8.51E-05 | 6.59E-05 | 1539 | 0.7948 | 0.8522 | 0.7948 |
| UTR3 | 3602 | 6.00E-04 | 1.50E-03 | 6.00E-04 | 2050 | 3.30E-03 | 6.00E-04 | 6.00E-04 | 1539 | 0.8977 | . | 0.8977 |
| Splicing | 3601 | 1.10E-03 | 1.65E-02 | 1.10E-03 | 2049 | 1.40E-03 | 7.40E-03 | 1.40E-03 | 1540 | 0.4740 | 0.7342 | 0.4740 |
| Frameshift Insertion | 3602 | 5.00E-03 | 1.20E-03 | 1.20E-03 | 2050 | 1.70E-03 | 2.85E-02 | 1.70E-03 | 1540 | 0.0165 | 0.0163 | 0.0163 |
| Intronic | 3602 | 1.08E-02 | 1.18E-01 | 1.08E-02 | 2050 | 1.07E-02 | 2.93E-02 | 1.07E-02 | 1539 | 0.0245 | 0.0049 | 0.0049 |
| Nonsynonymous | 3601 | 1.03E-01 | 4.67E-02 | 4.67E-02 | 2050 | 4.69E-02 | 7.21E-02 | 4.69E-02 | 1539 | 0.1829 | . | 0.1829 |
| Synonymous | 3601 | 2.53E-01 | 8.43E-02 | 8.43E-02 | 2049 | 6.38E-01 | 8.91E-02 | 8.91E-02 | 1539 | 0.0341 | 0.8191 | 0.0341 |
| Intergenic | 3602 | 0.0912 | 0.792 | 0.0912 | 2049 | 1.57E-01 | 7.40E-01 | 1.57E-01 | 1539 | 0.6496 | 0.9171 | 0.6496 |
| Nonframeshift Insertion | 3602 | 0.1805 | 0.0983 | 0.0983 | 2050 | 1.73E-01 | 7.77E-01 | 1.73E-01 | 1540 | 0.9277 | 0.1603 | 0.1603 |
| Stop gain | 3602 | 0.1256 | 0.6471 | 0.1256 | 2050 | 1.94E-01 | 2.27E-01 | 1.94E-01 | 1539 | 0.5117 | 0.681 | 0.5117 |
| Upstream | 3602 | 0.3445 | 0.1395 | 0.1395 | 2049 | 7.71E-01 | 2.58E-01 | 2.58E-01 | 1539 | 0.7667 | 0.2573 | 0.2573 |
| Nonframeshift deletion | 3602 | 0.1898 | 0.9897 | 0.1898 | 2050 | 4.46E-01 | 5.10E-01 | 4.46E-01 | 1539 | 0.8449 | 0.8944 | 0.8449 |
| ncRNA_exonic | 3602 | 0.7753 | 0.2866 | 0.2866 | 2050 | 4.64E-01 | 4.46E-01 | 4.46E-01 | 1539 | 0.1866 | 0.0976 | 0.0976 |
| Unknown | 3602 | 8.0E-01 | 0.4945 | 0.4945 | 2050 | 7.12E-01 | 4.43E-01 | 4.43E-01 | 1539 | 0.8680 | . | 0.868 |
| Frameshift Deletion | 3602 | 0.5398 | 0.8031 | 0.5398 | 2050 | 5.69E-01 | . | 5.69E-01 | 1539 | 0.4733 | 0.9911 | 0.4733 |
| UTR5 | 3602 | 0.6004 | 0.5713 | 0.5713 | 2050 | 6.49E-01 | 8.49E-01 | 6.49E-01 | 1539 | 0.5416 | 0.0877 | 0.0877 |
| Downstream | 3602 | 0.9159 | 0.7251 | 0.7251 | 2050 | 8.03E-01 | 9.13E-01 | 8.03E-01 | 1539 | 0.0143 | 0.4474 | 0.0143 |
| ncRNA_intronic | 3601 | 0.9983 | 0.9618 | 0.9618 | 2050 | 8.36E-01 | 9.74E-01 | 8.36E-01 | 1539 | 0.2103 | 0.3218 | 0.2103 |

*SNVs detected only in patients with MDD but not in 1000 Genomes or controls and consistently called by GATK and Varscan; .

P_log, p value for log-trasnformed SNV burden; P_nonlog, p value for non-log transformed; P_min, minumum p value of two.

**Table S14.** Post Hoc estimate of LS means of HAMD17 by time and genotype group

|  | |  |  | **Genotype** | |  |  | |  |  | |  |  | |
| --- | --- | --- | --- | --- | --- | --- | --- | --- | --- | --- | --- | --- | --- | --- |
| Gene | | SNPs | Time | **0** | **1** | **Beta** | **SE** | | **DF** | **t Value** | | **P** | **Adj P** | |
| Drug only | |  |  |  |  |  |  | |  |  | |  |  | |
| *IL1A* | | D289 |  |  |  |  |  | |  |  | |  |  | |
|  | | rs3783550 | Baseline | 3.171 | 3.167 | 0.005 | 0.0478 | | 2047 | 0.10 | | 0.9204 | 1 | |
|  | | DOM1189 | Week 2 | 2.503 | 2.467 | 0.036 | 0.0478 | | 2047 | 0.75 | | 0.4533 | 1 | |
|  | |  | Week 4 | 2.169 | 2.044 | 0.124 | 0.0478 | | 2047 | 2.60 | | 0.0094 | 0.3415 | |
|  | |  | Week 6 | 1.936 | 1.696 | 0.240 | 0.0479 | | 2047 | 5.02 | | 5.6E-07 | <.0001 | |
|  | |  | Week 8 | 1.775 | 1.510 | 0.265 | 0.0484 | | 2047 | 5.48 | | 4.8E-08 | <.0001 | |
| *IL1A* | |  |  |  |  |  |  | |  |  | |  |  | |
|  | | **rs3783553** | Baseline | 3.120 | 3.110 | 0.009 | 0.0477 | | 2047 | 0.19 | | 0.8493 | 1 | |
|  | | DOM2293 | Week2 | 2.570 | 2.520 | 0.043 | 0.0477 | | 2047 | 0.91 | | 0.3629 | 1 | |
|  | |  | Week4 | 2.250 | 2.110 | 0.136 | 0.0477 | | 2047 | 2.86 | | 0.0043 | 0.1759 | |
|  | |  | Week6 | 1.970 | 1.730 | 0.245 | 0.0478 | | 2047 | 5.12 | | 3.3E-07 | <.0001 | |
|  | |  | Week8 | 1.770 | 1.510 | 0.262 | 0.0483 | | 2047 | 5.41 | | 7.0E-08 | <.0001 | |
|  | |  |  |  |  |  |  | |  |  | |  |  | |
| *GNA15* | |  | Baseline | 3.119 | 3.134 | -0.014 | 0.1085 | | 2048 | -0.13 | | 0.8966 | 1 | |
|  | | rs11671393 | WEEK 2 | 2.500 | 2.454 | 0.045 | 0.1085 | | 2048 | 0.42 | | 0.6745 | 1 | |
|  | | REC15566 | WEEK 4 | 2.109 | 2.008 | 0.101 | 0.1085 | | 2048 | 0.93 | | 0.3525 | 1 | |
|  | |  | WEEK6 | 1.844 | 1.320 | 0.524 | 0.1085 | | 2048 | 4.83 | | 1.5E-06 | <.0001 | |
|  | |  | WEEK 8 | 1.679 | 1.002 | 0.677 | 0.1100 | | 2048 | 6.16 | | 8.7E-10 | <.0001 | |
| *PPP2CB* | |  |  |  |  |  |  | |  |  | |  |  | |
|  | | **rs4733201** | Baseline | 3.111 | 3.587 | -0.476 | 0.3839 | | 2048 | -1.24 | | 0.2151 | 1 | |
|  | | REC7900 | WEEK 2 | 2.487 | 3.101 | -0.614 | 0.3839 | | 2048 | -1.60 | | 0.1097 | 0.9945 | |
|  | |  | WEEK 4 | 2.092 | 2.836 | -0.744 | 0.3839 | | 2048 | -1.94 | | 0.0525 | 0.9114 | |
|  | |  | WEEK6 | 1.809 | 1.652 | 0.157 | 0.3839 | | 2048 | 0.41 | | 0.6818 | 1 | |
|  | |  | WEEK 8 | 1.644 | 0.128 | 1.516 | 0.3840 | | 2048 | 3.95 | | 8.1E-05 | 0.0036 | |
| *EPHA5* | |  |  |  |  |  |  | |  |  | |  |  | |
|  | | rs3749525 | Baseline | 3.110 | 3.268 | -0.158 | 0.2227 | | 2048 | -0.71 | | 0.4778 | 1 | |
|  | | REC4277 | WEEK 2 | 2.488 | 2.521 | -0.032 | 0.2227 | | 2048 | -0.15 | | 0.8808 | 1 | |
|  | |  | WEEK 4 | 2.091 | 2.368 | -0.277 | 0.2227 | | 2048 | -1.24 | | 0.2151 | 1 | |
|  | |  | WEEK6 | 1.813 | 1.316 | 0.498 | 0.2227 | | 2048 | 2.23 | | 0.0259 | 0.6863 | |
|  | |  | WEEK 8 | 1.645 | 0.708 | 0.937 | 0.2369 | | 2048 | 3.95 | | 8.1E-05 | 0.0036 | |
| Plus-rTMS treatment | | | |  |  |  |  | |  |  | |  |  | |
| *PLA2G4C*** | |  |  |  |  |  |  | |  |  | |  |  | |
|  | | **rs2303744** | Baseline | 3.158 | 3.166 | -0.009 | 0.0729 | | 1535 | -0.12 | | 0.9045 | 1 | |
|  | | REC16163 | WEEK 2 | 2.382 | 2.226 | 0.156 | 0.0729 | | 1535 | 2.14 | | 0.0325 | 0.7751 | |
|  | |  | WEEK 4 | 1.787 | 1.767 | 0.020 | 0.0729 | | 1535 | 0.27 | | 0.7872 | 1 | |
|  | |  | WEEK6 | 1.457 | 1.624 | -0.167 | 0.0732 | | 1535 | -2.28 | | 0.0227 | 0.6395 | |
|  | |  | WEEK 8 | 1.354 | 1.727 | -0.373 | 0.0753 | | 1535 | -4.96 | | 7.6E-07 | <.0001 | |
| *GBA*** | |  |  |  |  |  |  | |  |  | |  |  | |
|  | | **rs12034326** | Baseline | 3.159 | 3.105 | 0.054 | 0.1116 | | 1535 | 0.48 | | 0.6313 | 1 | |
|  | | REC932 | WEEK 2 | 2.351 | 2.295 | 0.056 | 0.1116 | | 1535 | 0.50 | | 0.6171 | 1 | |
|  | |  | WEEK 4 | 1.767 | 1.929 | -0.162 | 0.1116 | | 1535 | -1.46 | | 0.1444 | 0.9991 | |
|  | |  | WEEK6 | 1.458 | 1.829 | -0.371 | 0.1116 | | 1535 | -3.32 | | 9.1E-04 | 0.0399 | |
|  | |  | WEEK 8 | 1.378 | 1.947 | -0.570 | 0.1145 | | 1535 | -4.98 | | 6.9E-07 | <.0001 | |
| *GNA11* |  | |  |  |  |  |  |  | |  |  | | |  |
|  | **rs8092** | | Baseline | 3.160 | 3.130 | 0.030 | 0.1450 | 1535 | | 0.21 | 0.8337 | | | 1 |
|  | REC15556 | | WEEK 2 | 2.353 | 2.297 | 0.056 | 0.1450 | 1535 | | 0.38 | 0.7040 | | | 1 |
|  |  | | WEEK 4 | 1.793 | 1.464 | 0.329 | 0.1451 | 1535 | | 2.27 | 0.0233 | | | 0.6558 |
|  |  | | WEEK6 | 1.509 | 0.872 | 0.637 | 0.1451 | 1535 | | 4.39 | 1.2E-05 | | | 0.0005 |
|  |  | | WEEK 8 | 1.442 | 0.691 | 0.750 | 0.1604 | 1535 | | 4.68 | 3.1E-06 | | | 0.0001 |

Genotype, coded as 1 for homozygote of minor allele in recessive model and carrying of minor allele in dominant model; 0 otherwise;

**^**^**, one SNPs at *PLA2G4C* in LD was omitted here, to reduce the size of table and should show similar trend of effect with rs2303744;

Adj P, p value adjusted for multiple comparison, a conservative method to fit a heteroscedastic ANOVA model and perform Dunnett’s T3 method, which is based on the studentized maximum modulus (ADJUST=SMM).

**Table S15.** Top SNPs for modulating antidepressant response and a look up for genetic association with MDD.

|  |  |  |  |  |  |  |  | **Treatment response** | | | |  | **Association with risk of MDD**** | | | | | | | |
| --- | --- | --- | --- | --- | --- | --- | --- | --- | --- | --- | --- | --- | --- | --- | --- | --- | --- | --- | --- | --- |
| **Model** | **Chr** | **SNP** | **BP** | ***Gene*** | **Location** | **A1** | **A2** | **NDF** | **DDF** | **F** | **P** |  | **N** | **FA** | **FU** | **OR** | **SE** | **L95** | **U95** | **P** |
| **Drug-only group** | | |  |  |  |  |  |  |  |  |  |  |  |  |  |  |  |  |  |  |
| Dominant | 2 | rs3783553 | 113531715 | *IL1A* | UTR3 | TGAA | - | 4 | 2047 | 10.69 | 1.4E-08 |  | 1388 | 0.332 | 0.367 | 0.86 | 0.0883 | 0.72 | 1.02 | 0.0809 |
|  | 2 | rs3783550 | 113532885 | *IL1A* | Intronic | T | G | 4 | 2051 | 9.97 | 5.4E-08 |  | 1390 | 0.341 | 0.373 | 0.87 | 0.0862 | 0.74 | 1.04 | 0.1204 |
|  | 1 | rs3817192 | 204400650 | *PIK3C2B* | Intronic | C | A | 4 | 1996 | 8.60 | 7.0E-07 |  | 1345 | 0.416 | 0.453 | 0.87 | 0.0824 | 0.74 | 1.02 | 0.0843 |
|  | 2 | rs1609682 | 113540205 | *IL1A* | Intronic | T | G | 4 | 2047 | 8.30 | 1.2E-06 |  | 1384 | 0.336 | 0.370 | 0.87 | 0.0856 | 0.74 | 1.03 | 0.1039 |
|  | 2 | rs12695032 | 241815473 | *AGXT* | Intronic | A | G | 4 | 2048 | 7.98 | 2.2E-06 |  | 1391 | 0.278 | 0.302 | 0.89 | 0.0910 | 0.74 | 1.06 | 0.1906 |
|  | 17 | rs183190490 | 61557305 | *ACE* | Intronic | A | G | 4 | 2025 | 7.87 | 2.7E-06 |  | 1380 | 0.020 | 0.012 | 1.76 | 0.3619 | 0.86 | 3.57 | 0.1202 |
|  | 10 | rs2071426 | 96828323 | *CYP2C8* | Splicing | C | T | 4 | 2031 | 7.79 | 3.2E-06 |  | 1373 | 0.077 | 0.063 | 1.23 | 0.1623 | 0.90 | 1.70 | 0.1966 |
| Recessive | 19 | rs11671393 | 3151676 | *GNA15* | ncRNA_intronic | C | G | 4 | 2048 | 14.93 | 5.0E-12 |  | 1384 | 0.183 | 0.192 | 0.95 | 0.1027 | 0.77 | 1.16 | 0.5889 |
|  | 8 | rs4733201 | 30670390 | *PPP2CB* | Upstream | T | G | 4 | 2048 | 10.84 | 1.1E-08 |  | 1374 | 0.064 | 0.059 | 1.09 | 0.1720 | 0.78 | 1.53 | 0.6102 |
|  | 4 | rs3749525 | 66270241 | *EPHA5* | Intronic | G | A | 4 | 2048 | 8.59 | 7.1E-07 |  | 1389 | 0.122 | 0.096 | 1.31 | 0.1364 | 1.00 | 1.71 | 0.0469 |
|  | 10 | rs11006229 | 52350006 | *SGMS1* | UTR5 | T | C | 4 | 2048 | 8.17 | 1.6E-06 |  | 1386 | 0.091 | 0.108 | 0.82 | 0.1351 | 0.63 | 1.07 | 0.1506 |
|  | 10 | rs1810576 | 52220420 | *SGMS1* | Splicing | C | T | 4 | 2048 | 8.17 | 1.6E-06 |  | 1391 | 0.094 | 0.102 | 0.92 | 0.1388 | 0.70 | 1.21 | 0.5571 |
| **Plus-rTMS group** | | |  |  |  |  |  |  |  |  |  |  |  |  |  |  |  |  |  |  |
| Dominant | 1 | rs78810070 | 57176393 | *PRKAA2* | UTR3 | C | T | 4 | 1495 | 7.99 | 2.3E-06 |  | 1370 | 0.115 | 0.124 | 0.92 | 0.1269 | 0.72 | 1.18 | 0.5190 |
|  | 1 | rs10797428 | 2411451 | *PLCH2* | Intronic | A | G | 4 | 1531 | 7.92 | 2.6E-06 |  | 1391 | 0.204 | 0.212 | 0.97 | 0.1020 | 0.79 | 1.19 | 0.7645 |
|  | 1 | rs12727342 | 2409892 | *PLCH2* | Intronic | A | G | 4 | 1520 | 7.86 | 2.8E-06 |  | 1385 | 0.194 | 0.198 | 0.99 | 0.1040 | 0.81 | 1.21 | 0.9200 |
| Recessive | 19 | rs2303744 | 48602948 | *PLA2G4C* | Exonic | T | C | 4 | 1535 | 12.85 | 2.7E-10 |  | 1390 | 0.443 | 0.442 | 1.00 | 0.0830 | 0.85 | 1.18 | 0.9852 |
|  | 1 | rs9628662 | 155206341 | *GBA* | Intronic | T | G | 4 | 1535 | 11.67 | 2.4E-09 |  | 1375 | 0.234 | 0.244 | 0.95 | 0.0947 | 0.79 | 1.15 | 0.6069 |
|  | 1 | rs12034326 | 155214473 | *GBA* | UTR5 | A | G | 4 | 1535 | 10.12 | 4.4E-08 |  | 1386 | 0.235 | 0.240 | 0.97 | 0.0947 | 0.81 | 1.17 | 0.7694 |
|  | 19 | rs1653554 | 48608472 | *PLA2G4C* | Intronic | G | A | 4 | 1535 | 8.79 | 5.1E-07 |  | 1375 | 0.391 | 0.393 | 1.00 | 0.0837 | 0.85 | 1.17 | 0.9632 |
|  | 2 | rs7557421 | 46207618 | *PRKCE* | Intronic | A | G | 4 | 1535 | 8.70 | 6.0E-07 |  | 1385 | 0.110 | 0.113 | 0.99 | 0.1257 | 0.77 | 1.26 | 0.9091 |
|  | 17 | rs9911574 | 78795868 | *RPTOR* | Intronic | G | A | 4 | 1535 | 7.94 | 2.5E-06 |  | 1371 | 0.136 | 0.152 | 0.89 | 0.1151 | 0.71 | 1.11 | 0.3008 |
|  | 19 | rs8092 | 3123635 | *GNA11* | UTR3 | T | C | 4 | 1535 | 7.87 | 2.8E-06 |  | 1391 | 0.176 | 0.228 | 0.73 | 0.1005 | 0.60 | 0.88 | 0.0014 |

Drug-only, individuals treated with pharmacological treatment only; plus-rTMS, individuals treated with pharmacological drug plus rTMS in the first two weeks; Only SNPs with minor allele frequency>5% were included for analysis; All SNPs were in Hardy-Weinberg equilibrium (P>0.01); NDF, numerator degree of freedom; DDF, denominator degree of freedom; FA, FU, minor allele frequency in patients and normal controls, respectively; **, association analysis from our case-control.

**Table S16**. *cis*-eQTL for loci at genome-wide significance for antidepressant response

| SNP | Gene | Effect allele | NES | P** | Top tissue | Other tissues |  |
| --- | --- | --- | --- | --- | --- | --- | --- |
| rs3783550 | *IL1A* | Major G | 0.30 | 4.9x10^-05^ | Skin | Pituitary |  |
| rs4733201 | *PPP2CB* | Minor T | 0.83 | 1x10^-39^ | Whole blood |  |  |
| rs1653554 | *PLA2G4C* | Minor G | 0.53 | 2.5x10^-24^ | Tibial nerve | Adipose, whole blood |  |
| rs12034326 | *GBA* | Minor T | -0.57 | 3.00x10^-49^ | Esophagus | Whole blood |  |
| rs8092^*^ | *GNA15* | Major C | >0 | 6x10^-04^ | Prefrontal cortex |  |  |

Note: Most of analysis except rs8092 was based on online analysis of the genotype-tissue expression (GTEx)dataset;

*, Analysis performed based on an independent mRNA expression data of prefrontal cortex of postmortem human brain in the BrainClould dataset;

**, FDR<0.05.

**Table S17**. Replication of loci reported in previous pharmacogenomic study(P<0.05)

|  | |  | | |  |  | |  |  |  |  | Treatment response | | | |  |
| --- | --- | --- | --- | --- | --- | --- | --- | --- | --- | --- | --- | --- | --- | --- | --- | --- |
|  | | **Chr** | | | **SNP** | **BP** | | **Gene** | **Region** | **A1** | **A2** | **NDF** | **DDF** | **F** | **P** |  |
| **Dominant Model** | | | | | |  | |  |  |  |  |  |  |  |  |  |
| Drug-only | | | | |  |  | |  |  |  |  |  |  |  |  |  |
|  | | | 7 | | rs2188524 | 87230435 | | *ABCB1* | intronic | C | T | 4 | 2049 | 2.93 | 1.99E-02 |  |
|  | | | 11 | | rs727155 | 27750449 | | *BDNF* | intergenic | T | C | 4 | 2045 | 3.09 | 1.50E-02 |  |
|  | | | 10 | | rs17885098 | 96522561 | | *CYP2C19* | exonic | C | T | 4 | 2054 | 2.70 | 2.90E-02 |  |
|  | | | 10 | | rs4244285 | 96541616 | | *CYP2C19* | exonic | A | G | 4 | 2003 | 3.09 | 1.50E-02 |  |
|  | | | 22 | | rs16947 | 42523943 | | *CYP2D6* | exonic | A | G | 4 | 2057 | 2.40 | 4.80E-02 |  |
|  | | | 16 | | rs2279805 | 55729124 | | *SLC6A2* | intronic | T | C | 4 | 2050 | 2.93 | 1.99E-02 |  |
|  | | | 16 | | rs36009 | 55732620 | | *SLC6A2* | intronic | T | C | 4 | 2015 | 2.82 | 2.40E-02 |  |
|  | | | 16 | | rs73544099 | 55707269 | | *SLC6A2* | intronic | T | C | 4 | 2054 | 3.97 | 3.25E-03 |  |
| plus-rTMS | | | | |  |  | |  |  |  |  |  |  |  |  |  |
|  | | | 7 | | rs10276036 | 87180198 | | *ABCB1* | intronic | T | C | 4 | 1488 | 2.74 | 2.72E-02 |  |
|  | | | 7 | | rs1128503 | 87179601 | | *ABCB1* | exonic | G | A | 4 | 1535 | 2.94 | 1.97E-02 |  |
|  | | | 7 | | rs1202168 | 87195962 | | *ABCB1* | intronic | G | A | 4 | 1417 | 3.73 | 5.06E-03 |  |
|  | | | 7 | | rs1922240 | 87183354 | | *ABCB1* | intronic | C | T | 4 | 1361 | 2.84 | 2.31E-02 |  |
|  | | | 7 | | rs2235033 | 87179143 | | *ABCB1* | intronic | G | A | 4 | 1535 | 2.73 | 2.80E-02 |  |
|  | | | 7 | | rs2235046 | 87174066 | | *ABCB1* | intronic | C | T | 4 | 1531 | 2.68 | 3.05E-02 |  |
| **Recessive model** | | | | | |  |  | |  |  |  |  |  |  |  |  |
| Drug-only | | | |  | |  |  | |  |  |  |  |  |  |  |  |
|  | 16 | | | rs168924 | | 55689544 | *SLC6A2* | | UTR5 | G | A | 4 | 2048 | 2.83 | 2.35E-02 |  |
|  | 16 | | | rs2279805 | | 55729124 | *SLC6A2* | | intronic | T | C | 4 | 2049 | 2.45 | 4.40E-02 |  |
| plus-rTMS | | | | | |  |  | |  |  |  |  |  |  |  |  |
|  | 13 | | | rs6311 | | 47471478 | *HTR2A* | | upstream | C | T | 4 | 1535 | 3.05 | 1.61E-02 |  |
|  | 13 | | | rs6313 | | 47469940 | *HTR2A* | | exonic | G | A | 4 | 1535 | 3.13 | 1.41E-02 |  |
|  | 13 | | | rs7997012 | | 47411985 | *HTR2A* | | intronic | A | G | 4 | 1535 | 4.24 | 2.04E-03 |  |
|  | 16 | | | rs168924 | | 55689544 | *SLC6A2* | | UTR5 | G | A | 4 | 1535 | 3.33 | 1.01E-02 |  |
|  | 16 | | | rs2242447 | | 55735912 | *SLC6A2* | | intronic | C | T | 4 | 1535 | 2.81 | 2.42E-02 |  |
|  | 16 | | | rs2279805 | | 55729124 | *SLC6A2* | | intronic | T | C | 4 | 1534 | 3.07 | 1.58E-02 |  |
|  | 16 | | | rs36023 | | 55707254 | *SLC6A2* | | intronic | A | G | 4 | 1535 | 3.06 | 1.60E-02 |  |
|  | 16 | | | rs73544099 | | 55707269 | *SLC6A2* | | intronic | T | C | 4 | 1535 | 6.85 | 1.83E-05 |  |

Drug-only, individuals treated with pharmacological treatment only; Plus-rTMS, individuals treated with pharmacological drug plus rTMS in the first two weeks; Only SNPs with minor allele frequency>5% were included for analysis; All SNPs were in Hardy-Weinberg equilibrium (P>0.01); NDF, numerator degree of freedom; DDF, denominator degree of freedom.

**2. Supplementary figures**

**Figure S1. Flow chart of the study sample**

Recruited subjects N=1840

(1,331 cases, 509 controls)

MDD subjects excluded for inconsistent diagnose got from two psychiatrists, N=79

Subjects excluded due to unavailability of DNA samples, N=77

MDD subjects excluded for non-compliance, drop-out or change of drug, N=133

Control subjects excluded for getting depression during 12 months follow up, N=1

MDD patients excluded due to electroconvulsive therapy received during study, N=20

MDD subjects excluded with change of diagnosis during 12 months follow-up, N=33(Bipolar 9, Schizophrenia 10, others N=14)

Samples genotyped N=1,497 (999 cases and 498 controls)

Analysis QC removed 19 individuals with missing genotypes, no evident outlier was detected for removal

Subjects excluded for variants with low calling quality (<30) or depth coverage lower than 5x, N=111

Subjects entering statistic analyses N=1,367 (929 MDD cases and 438 controls)

**Figure. S2. QQ plots of P values for SNPs modulating antidepressant response.**

**A)** Dominant model in all patients together adjusted for treatment group; **B).** Dominant model in the antidepressant drug-only therapy; **C)** Dominant model in the plus-rTMS therapy**; D)**. Recessive model in all patients together adjusted for treatment group; E**)**. Recessive model in the antidepressant drug-only therapy; **F)**. Recessive model in the plus-rTMS therapy


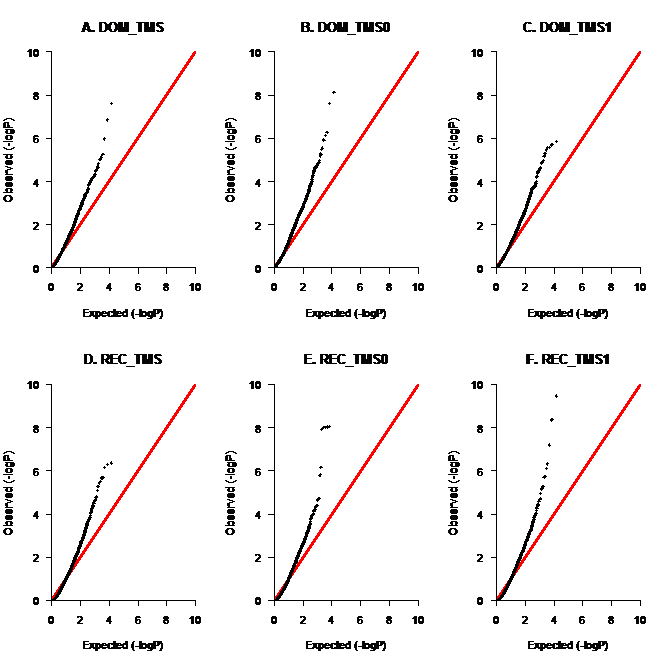


**Figure. S3. Manhattan plots of P values for SNPs modulating antidepressant response. A)** Dominant model in all patients together adjusted for treatment group; **B)** Dominant model in the antidepressant drug-only therapy; **C)** Dominant model in the plus-rTMS therapy**; D)** Recessive model in all patients together adjusted for treatment group; **E)** Recessive model in the antidepressant drug-only therapy; **F)** Recessive model in the plus-rTMS therapy.


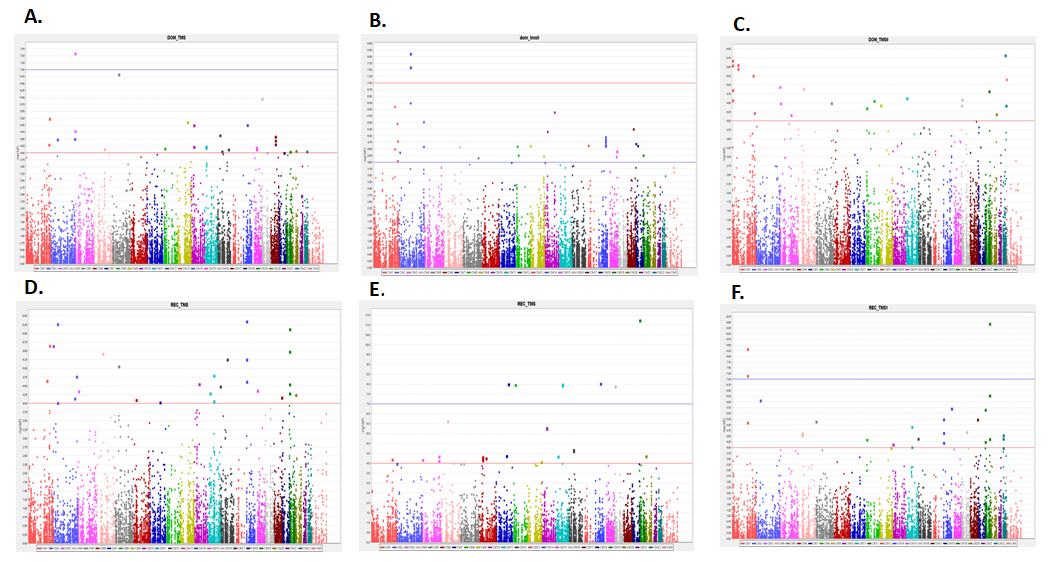


**Figure. S4. SNP rs8092 *cis*-effect in human tissue of thyroid**. Major risk allele increased the expression of *GNA11* in thyroid (NES=-0.12 and P=2.3x10^-05^) (www.gtexprtal.org)


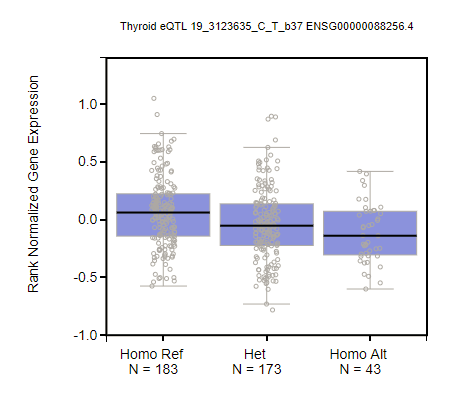


**3. Supplementary methods**

**Methods 1. Sample size determination for genetic study of antidepressant response.**

The calculation of sample size was performed based on the repeated measures ANOVA. The following inputs were used to compute sample size 1) type I error was specified as 0.000006 considering subgroup analysis; 2) predictors was the genotype group, effect allele frequency was 0.3 for dominant model and 0.7 for recessive model; 3) the test hypothesis was that antidepressant response was different between genotype group; 4) assuming means of log-transformed HAM-D17 in one genotype group, and 10% more effective in the other genotype group after treated more than 4 weeks (**Table SM1**); 5) Correlation matrix was also determined from previous results (**Table SM2**); 6) standard deviation: 0.6 and 0.7, estimates from the clinical data.

**Table SM1.** Parameters for the means of logHAMD17 over time by genotype group

| Genotype | Baseline | Week 2 | Week 4 | Week 6 | Week8 |  |
| --- | --- | --- | --- | --- | --- | --- |
| Group 1 | 3.13 | 2.78 | 2.07 | 1.80 | 1.45 |  |
| Group 0 | 3.13 | 2.78 | 2.30 | 2.015 | 1.61 |  |

Note: Overall treatment effect was obtained from a large antidepressant trial for 8 weeks^1^.

**Table SM2.** Parameters for conjectured correlation matrix used for sample size calculation for genetic study of antidepressant effectiveness

|  | Baseline | Week 2 | Week 4 | Week 6 | Week 8 |
| --- | --- | --- | --- | --- | --- |
| Baseline | 1 | 0.48 | 0.35 | 0.26 | 0.22 |
| Week 2 | 0.48 | 1 | 0.48 | 0.51 | 0.36 |
| Week 4 | 0.35 | 0.71 | 1 | 0.35 | 0.57 |
| Week 6 | 0.26 | 0.51 | 0.77 | 1 | 0.77 |
| Week 8 | 0.22 | 0.36 | 0.57 | 0.77 | 1 |

Note: calculated from all patients in this study

**Figure SM1**. Plot of sample size required for detecting an effect of genotype on antidepressant response (+, plots for sample requited to detect time and genotype interaction).

**
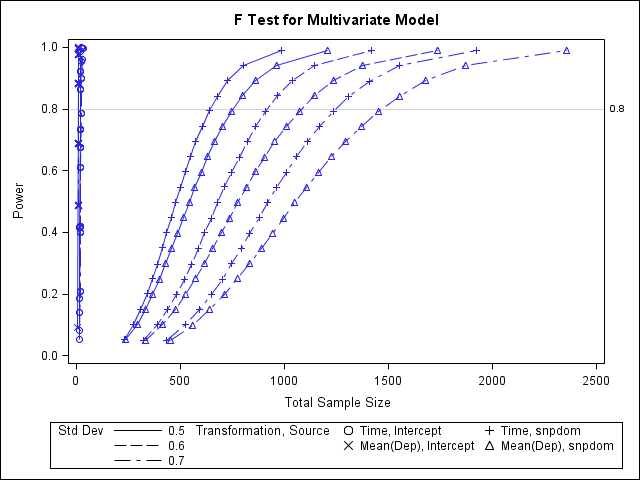
**

**Table SM3.** List of pathways or system to which genes were selected

| Pathway |  | Number of gene | BP |
| --- | --- | --- | --- |
| Monoaminergic pathway |  | 188 | 863692 |
| Glutamatergic system |  | 111 | 652941 |
| Gamma amino butyric acid (GABA)-energic system |  | 67 | 390060 |
| BDNF system |  | 97 | 497504 |
| mTOR signaling pathway |  | 75 | 439094 |
| Neuroplasticity related genes |  | 46 | 304490 |
| Long-term potentiation |  | 67 | 406625 |
| Hypothalamic–pituitary–adrenal axis |  | 18 | 70704 |
| Immune system |  | 70 | 203418 |
| G-protein linked signaling system |  | 31 | 113565 |
| Glycine pathway |  | 31 | 94446 |
| Cannabinoid system |  | 82 | 412176 |
| Purine system |  | 17 | 55618 |
| Estrogen system |  | 93 | 493145 |
| Folate acid system |  | 31 | 101014 |
| Renin-angiotensin |  | 14 | 52842 |
| Melanocortin receptor |  | 6 | 12515 |
| Melatonin related genes |  | 61 | 192512 |
| Leptin system |  | 2 | 11668 |
| Circadian Rhythms System |  | 24 | 124909 |
| Notch signaling pathway |  | 29 | 146153 |
| Cytochrome P450 genes |  | 11 | 38214 |
| White matter lesions related genes |  | 13 | 75964 |
| Potassium channel genes |  | 16 | 46598 |
| Glutamate- NMDA-CaM signaling |  | 9 | 43297 |
| PLC-DAG-PKC-CREB |  | 33 | 229847 |
| Other depression-related genes |  | 40 | 169567 |
| Genes identified from recent GWAS |  | 89 | 421625 |

**Table SM4**. List of genes selected for target exome-sequencing

| Pathway | List of Genes | Number of genes | BP |
| --- | --- | --- | --- |
| Monoaminergic  pathway | *HTR1A, HTR1B, HTR1D, HTR1E, HTR1F, HTR2A, HTR2B, HTR2C, HTR3A, HTR3B, HTR3C, HTR3D, HTR3E, HTR4, HTR5A, HTR6, HTR7, SLC6A3, SLC6A4, SLC18A1, SLC18A2, TPH1, TPH2, PLCB1, PLCB2, PLCB3, PLCB4, ITPR1, ITPR2, ITPR3, TRPC1, CACNA1A, CACNA1B, CACNA1C, CACNA1D, CACNA1F, CACNA1S, GNAQ, PRKCA, PRKCB, PRKCG, MAPK1, MAPK3, PLA2G4A, PLA2G4B, PLA2G4C, PLA2G4D, PLA2G4E, PLA2G4F, JMJD7-PLA2G4B, CYP2C18, CYP2C19, CYP2C8, CYP2C9, CYP2D6, CYP2J2, CYP4X1, ALOX12, ALOX5, ALOX12B, ALOX15, ALOX15B, PTGS1, PTGS2, ADCY5, PRKACA, PRKACB, PRKACG, PRKX, RAPGEF3, KCND2, GABRB1, GABRB2, GABRB3, GNAI1, GNAI2, GNAI3, GNAO1, GNB1, GNB2, GNB3, GNB4, GNB5, GNG2, GNG3, GNG4, GNG5, GNG7, GNG8, GNG10, GNG11, GNG12, GNG13, GNGT1, GNGT2, CASP3, DUSP1, HRAS, ARAF, BRAF, RAF1, MAP2K1, KCNJ3, KCNJ5, KCNJ6, KCNJ9, APP, ADRA1A, ADRA1B, ADRA1D, ADRA2A, ADRA2B, ADRA2C, ADRB1, ADRB2, ADRB3, COMT , MAOA, MAOB, DRD1, DRD2, DRD3, DRD4, DRD5, TH, DDC, CALY, CALML3, CALML5, CALML6, CALM1, CALM2, CALM3, CAMK2A, CAMK2B, CAMK2D, CAMK2G, PPP3CA, PPP3CB, PPP3CC, GNAS, CREB1, CREB3, CREB3L1, CREB3L2, CREB3L3, CREB3L4, CREB5, ATF2, ATF4, ATF6B, MAPK11, MAPK12, MAPK13, MAPK14, FOS, PPP1CA, PPP1CB, PPP1CC, PPP1R1B, SCN1A, PPP2CA, PPP2CB, ARRB2, PPP2R1A, PPP2R1B, PPP2R2A, PPP2R2B, PPP2R2C, PPP2R2D, GRIN2A, GRIN2B, GRIA1, GRIA2, GRIA3, GRIA4, KIF5A, KIF5B, KIF5C, AKT1, AKT2, AKT3, GSK3A, GSK3B, ARNTL, CLOCK* | 188 | 863692 |
| Glutamatergic  system | *GRIA1, GRIA2, GRIA3, GRIA4, GRID2, GRIK1, GRIK2, GRIK3, GRIK4, GRIK5, GRIN1, GRIN2A, GRIN2B, GRIN2C, GRIN2D , GRIN3A, GRIN3B, GRM1, GRM2, GRM3, GRM4, GRM5, GRM6, GRM7, GRM8, TPRC1, TPRC3, TPRC4, TPRC5, TPRC6, SLC38A1, SLC38A2, SLC38A3, SLC1A1, SLC1A2, SLC1A3, SLC1A6, SLC1A7, SLC17A6, SLC17A7, SLC17A8, ADRBK1, ADRBK2, ADCY1, ADCY2, ADCY3, ADCY4, ADCY5, ADCY6, ADCY7, ADCY8, ADCY9, GNAQ, GNAI1, GNAI2, GNAI3, GNAO1, GNB1, GNB2, GNB3, GNB4, GNB5, GNG2, GNG3, GNG4, GNG5, GNG7, GNG8, GNG10, GNG11, GNG12, GNG13, GNGT1, GNGT2, KCNJ3, CACNA1A, CACNA1C, CACNA1D, DLG4, DLGAP1, SHANK1, SHANK2, SHANK3, HOMER1, HOMER2, HOMER3, PLCB1, PLCB2, PLCB3, PRKCA, PRKCB, PRKCG, ITPR1, ITPR2, ITPR3, PLA2G4A, PLA2G4B, PLA2G4C, PLA2G4D, PLA2G4E, PLA2G4F, JMJD7-PLA2G4B, PLD1, PLD2, MAPK1, MAPK3, PRKACA, PRKACB, PRKACG, PRKX, PPP3CA, PPP3CB, PPP3CC, PPP3R1, PPP3R2* | 111 | 652941 |
| Gamma amino butyric acid (GABA)-energic system | *GABRA1, GABRA2, GABRA3, GABRA4, GABRA5, GABRA6, GABRB1, GABRB2, GABRB3, GABRD, GABRE, GABRG1, GABRG2, GABRG3, GABRP, GABRQ, GABRR1, GABRR2, GABRR3, GABBR1, GABBR2, GABARAP, GABARAPL1, GABARAPL2, NSF, HAP1, TRAK2, SRC, PLCL1, GLUL, ABAT, GLS, GLS2, GAD1, GAD2, SLC6A1, SLC6A11, SLC6A13, SLC12A5, SLC38A1, SLC38A2, SLC38A3, SLC38A5, SLC32A1, CACNA1A, CACNA1B, CACNA1C, CACNA1D, CACNA1F, CACNA1S, PRKCA, PRKCB, PRKCG, ADCY1, ADCY2, ADCY3, ADCY4, ADCY5, ADCY6, ADCY7, ADCY8, ADCY9, PRKACA, PRKACB, PRKACG, PRKX, KCNJ6* | 67 | 390060 |
| BDNF system | *SERPINE1, BDNF, NTF3, NTF4, NGFR, NTRK1, NTRK2, NTRK3, KIDINS220, FRS2, CRK, CRKL, RAPGEF1, RAP1A, RAP1B, RAF1 , BRAF, MAP3K3, MAP2K1, MAP2K2, MAP2K5, MAPK1, MAPK3, MAPK7, MAPKAPK2, RPS6KA1, RPS6KA2, RPS6KA3, RPS6KA5, RPS6KA6, BCL2, SH2B1, SH2B2, SH2B3, GRB2, SOS1, SOS2, HRAS, ABL1, PTPN11, SHC1, SHC2, SHC3, SHC4, GAB1, IRS1, MATK, PIK3CA, PIK3CB, PIK3CD, PIK3CG, PIK3R1, PIK3R2, PIK3R3, PIK3R5, PDK1, AKT1, AKT2, AKT3, NFKBIB, NFKB1, FASLG, BAD, GSK3B, PLCG1, PLCG2, PRKCD, CALM1P1, CALM1, CALML3, CALML5, CALML6, CAMK2A, CAMK2B, CAMK2D, CAMK2G, CAMK4, ATF4, PSEN1, PSEN2, ARHGDIA, ARHGDIB, ARHGDIG, CARF, FOXO1, FOXO3, FOXO4, FOXO6, CREBBP, CREM, MAPK4, MAPK6, MAPK15, MAP2K3, MAP2K6* | 97 | 497504 |
| mTOR signaling pathway | *CACNA1C, CACNA1D, CACNA1F, CACNA1S , INS, IGF1, IRS1, PIK3CA, PIK3CB, PIK3CD, PIK3CG, PIK3R1, PIK3R2, PIK3R3, PIK3R5, PTEN, PDPK1, AKT1, AKT2, AKT3, AKT1S1, NTRK2, GRB2, HRAD, KRAS, NRAS, RAF1, MAP2K1, MAP2K2, MAPK1, MAPK3, PRKCA, PRKCB, PRKCG, MLST8, MTOR, RICTOR, RPTOR, RPS6KA1, RPS6KA2, RPS6KA3, RPS6KA6, RPS6KB1, RPS6KB2, TSC1, TSC2, RHEB, HIF1A, EIF4EBP1, ULK1, ULK2, ULK3, RRAGA, RRAGB, RRAGC, RRAGD, VEGFA, EIF4B, EIF4E, RPS6, DDIT4, TNF, IKBKB, PRKAA1, PRKAA2, STK11, STRADA, CAB39, CAB39L, BRAF, DLG4, ARC, GRM1, SYN1* | 75 | 439094 |
| Neuroplasticity related genes | *BDNF, NGFR, SERPINE, DTNBP1, SYP, ARRB1, GAP43, CCL5, ENDOD1, ENPP2, VEGFA, VEGFB, FLT1, GABRA4, SPRY2, KCND2, NR3C1, NRN1, NPTX2, KLF10, ANKRD1, ARL4D, NPAS4, SERPINI1, RELN, ITGB1, ITGA2, LRP8, VLDLR, ITGA3, IGF1, L1CAM, DLG4, CARF, LAMA1, LAMA2, LAMA3, LAMA4, LAMA5, LAMB1, LAMB2, LAMB3, LAMB4, LAMC1, LAMC2, LAMC3* | 46 | 304490 |
| Long-term potentiation | *GRIA1, GRIA2, ADCY1, ADCY8, GRIN1, GRIN2A, GRIN2B, GRIN2C, GRIN2D, CACNA1C, GRM1, GRM5, GNAQ, PLCB1, PLCB2, PLCB3, PLCB4, ITPR1, ITPR2, ITPR3, PRKCA, PRKCB, PRKCG, CALM1, CALM2, CALM3, CALML3, CALML5, CALML6, HRAS, KRAS, NRAS, ARAF, BRAF, RAF1, MAP2K1, MAP2K2, MAPK1, MAPK3, RPS6KA1, RPS6KA2, RPS6KA3, RPS6KA6, ATF4, CREBBP, EP300, CAMK4, PPP3CA, PPP3CB, PPP3CC, PPP3R1, PPP3R2, PPP1CA, PPP1CB, PPP1CC, PPP1R1A, CAMK2A, CAMK2B, CAMK2D, CAMK2G, RAP1A, RAP1B, RAPGEF3, PRKACA, PRKACB, PRKACG, PRKX* | 67 | 406625 |
| Hypothalamic–pituitary–adrenal axis | *PRKACB, PRKACG, UCN2, UCN3, CRH, CRHR1, CRHR2, CRHRBP, AVPR1A, AVPR1B, AVPR2, NR3C1, BAG1, FKBP5, FKBP4, STUB1, PTGES3, POMC* | 18 | 70704 |
| Immune system | *INFR1, INFG, TNF, TNFRSF1A, IL1A, IL1R1, IL1R2, IL1RAP, IL1B, IL2, IL4, IL6, IL6R, IL6ST, CXCL8, IL10, IL10RA, IL10RB, IL11, IL11RA, IL12A, IL12B, IL13, IL18, IL18BP, IL20, IL24, IL28, IFNL3, PLA2G1B, COX2, MPO, NOSs, LTA4H, VWF, VCAM1, PTGER2, IDO1, IDO2, ABCB1, CD3E, CD4, CD7, CRH, CRHBP, CRHR1, CRHR2, CYP3A4, IPO13, JUND, MFNG, NR3C1, FKBP4, GTF2F1, POMC, RAC2, CDC42SE2, UCN, UCN2, UCN3, CCL2, GLYATL1, PSMB4, TBX2, PRKCSH, PSMD9, STAT1, STAT3, CRP* | 70 | 203418 |
| G-protein linked signaling system | *GNAI1, GNAI3, GNAO1, GLYATL1, GNAZ, GNAS, GNAL, GNAQ, GNA11, GNA12, GNA13, GNA14, GNA15, GNB1, GNB2, GNB3, GNB4, GNB5, GNG2, GNG3, GNG4, GNG5, GNG7, GNG8, GNG10, GNG11, GNG12, GNG13, GNGT1, GNGT2* | 31 | 113565 |
| Glycine pathway | *SHMT1, SHMT2, SLC6A9, SLC6A5, AMT, DLD, GCSH, GLDC, SLC32A1, GLRA1, GLRA2, GLRA3, GLRA4, GLRB, AGXT, AGXT2, DAO, GGT1, AOAT2, ALAS1, ALAS2, GAMT, GATM, PIPOX, SAROH, GNMT, GCAT, AOC2, AOC3, MAOA, MAOB* | 31 | 94446 |
| Cannabinoid system | *CNR1, CNR2, FAAH, MGLL, ABHD6, DAGLA, DAGLB, COX2, LPAR6, COX2, CACNA1A, CACNA1B, CACNA1C, CACNA1D, CACNA1F, CACNA1S, GRIA1, GRIA2, GRIA3, GRIA4, GRM1, GRM5, GNAQ, GNAI1, GNAI2, GNAI3, GNAO1, GNB1, GNB2, GNB3, GNB4, GNB5, GNG2, GNG3, GNG4, GNG5, GNG7, GNG8, GNG10, GNG11, GNG12, GNG13, GNGT1, GNGT2, PLCB1, PLCB2, PLCB3, PLCB4, ITPR1, ITPR2, ITPR3, PRKACA, PRKACB, PRKACG, PRKX, ADCY1, ADCY2, ADCY3, ADCY4, ADCY5, ADCY6, ADCY7, ADCY8, ADCY9, PRKCA, PRKCB, PRKCG, KCNJ3, KCNJ5, KCNJ6, KCNJ9, MAPK1, MAPK3, MAPK8, MAPK9, MAPK10, MAPK11, MAPK12, MAPK13, MAPK14, SLC32A1, RIMS1* | 87 |  |
| Purine system | *P2RY1, P2RY2, P2RY4, LPAR6, P2RY6, P2RY10, P2RY11, P2RY12, P2RY13, P2RY14, P2RX1,*  *P2RX2, P2RX3, P2RX4, P2RX5, P2RX6, P2RX7* | 17 | 55618 |
| Estrogen system | *ESR1, ESR2, GPER1, HSP90AA1, HSP90AB1, HSP90B1, HSPA1A, HSPA1B, HSPA1L, HSPA2, HSPA6, HSPA8, FKBP4, FKBP5, FOS, JUN, SP1, MMP2, MMP9, SRC, GNAS, HBEGF, PRKACA, PRKACB, PRKACG, PRKX, ADCY1, ADCY2, ADCY3, ADCY4, ADCY5, ADCY6, ADCY7, ADCY8, ADCY9, EGFR, SHC1, SHC2, SHC3, SHC4, GRB2, SOS1, SOS2, HRAS, NRAS, KRAS, RAF1, MAPK2K1, MAPK2K2, MAPK1, MAPK3, CREB1, CREB3, CREB3L1, CREB3L2, CREB3L3, CREB3L4, CREB5, ATF2, ATF4, ATF6B, CREBL2, CREBL3, CREBL4, PIK3R1, PIK3R2, PIK3R3, PIK3R5, PIK3CA, PIK3CB, PIK3CD, PIK3CG, PRKCD, GNAQ, PLCB1, PLCB2, PLCB3, PLCB4, ITPR1, ITPR2, ITPR3, GRM1, CALM1, CALM2, CALM3, CALML3, CALML5, CALML6, AKT1, AKT2, AKT3, NOS3* | 93 | 493145 |
| Folate acid system | *SHMT1, SHMT2, MTHFR, MTR, TCN2, MTR, BHMT, CBS, FOLR1, FOLR2, FOLR3, SLC19A1, DHFR, DHFRL1, AMT, ATIC, GART, MTHFD1L, MTHFD1, MTHFD2, MTHFD2L, THFS, FOLD, ADH1L1, ADH1L2, MTFMT, FTCD, TYMS, THY1, THY2* | 31 | 101014 |
| Renin-angiotensin | *AGT, REN, ACE, ACE2, AGTR1, AGTR2, CTSA, CTSG, CPA3, CMA1, MME, ENPEP, ANPEP, LNPEP* | 14 | 52842 |
| Melanocortin   receptor | *POMC, MC1R, MC2R, MC3R, MC4R, MC5R* | 6 | 12515 |
| Melatonin related genes | *TPH1, TPH2, DDC, AANAT, ASMT, DIT2, IDO1, IDO2, INMT, MAOA, MAOB, TDO2, tnaA, AFMID, KYNU, KMO, CYP79B2, CYP79B3, TAAR1, MTNR1A, MTNR1B, GPR50, PRKCA, PRKCB, PRKCG, GNAI1, GNAI2, GNAI3, GNAO1, GNB1, GNB2, GNB3, GNB4, GNB5, GNG2, GNG3, GNG4, GNG5, GNG7, GNG8, GNG10, GNG11, GNG12, GNG13, GNGT1, GNGT2, GNAQ, PLCB1, PLCB2, PLCB3, PLCB4, KCNJ3, KCNJ5, KCNJ6, KCNJ9* | 61 | 192512 |
| Leptin system | *LEP, LEPR* | 2 | 11668 |
| Circadian Rhythms System | *Per1, Per2, Per3, ARNTL, ARNTL2, CSNK1D, CSNK1E, CRY1, CRY2, NRID1, CLOCK, NPAS2 , RORA, RORB, RORC, RBX1, CUL1, SKP1, FBXW11, BTRC, FBXL3, BHLHE40, BHLHE41, TEF* | 24 | 124909 |
| Notch signaling pathway | *DLL1, DLL3, DLL4, JAG1, JAG2, NOTCH1, NOTCH2, NOTCH3, NOTCH4, LFNG, MFNG, RFNG, DVL1, DVL2, DVL3, NUMB, NUMBL, DTX1, DTX2, DTX3, DTX4, DTX3L, ADAM17, PSENEN, PSEN1, PSEN2, NCSTN, APH1A, APH1B* | 29 | 146153 |
| Cytochrome P450 genes | *CYP1A2, CYP2A6, CYP2C19, CYP2C9, CYP2D6, CYP2E1, CYP2B6, CYP2C8, CYP3A4, ABCB1, ABCC1* | 11 | 38214 |
| White matter lesions related genes | *CLDN11, CNP, DISC1, GSN, MAG, MAL, MBP, MOG, NRG1, OLIG1, OLIG2, QKI, TF* | 13 | 75964 |
| Potassium channel genes | *KCNK1, KCNK2, KCNK3, KCNK4, KCNK5, KCNK6, KCNK7, KCNK9, KCNK10, KCNK12, KCNK13, KCNK15, KCNK16, KCNK17, KCNK18* | 16 | 46598 |
| Glutamate- NMDA-CaM signaling | *CaMK1D, CaMK1G, CaMK2A, CaMK2B, CaMK2D, CaMK2g, CaMK4, ITPKB, ITPKC* | 9 | 43297 |
| PLC-DAG-PKC-CREB | *ADCY2, ADCY3, ADCY7, ADCY8, ADCY9, ADCY10, NTRK2, AKT1, AKT2, PIK3C2A, PIK3C2B, PIK3R5, PRKCA, PRKCB, PRKCD, PRKCE, PRKCG, PRKCH, PRKCQ, PRKCZ, PLCB1, PLCB2, PLCB3, PLCB4, PLCD1, PLCD3, PLCD4, PLCE1, PLCG1, PLCG2, PLCH1, PLCH2, PNCK* | 33 | 229847 |
| Other depression-related genes | *CHRNA7, DAOA, PRODH, S100A10, GDNF, GFRA1, GFRAL, FRK, SERTAD1, TSPAN1, APOA4, APOE, DAG1, DBH, CUX2, TAAR6, VAMP2, HDAC5, HES6, s100B, ADM, GCHFR, FOXO1, SGK1, CDKN1B, CDKN1C, ARRB2, GADD45B, MCTP2, ENPP2, ARRB1, DDX60, DDX60L, OPRM1, REEP1, REEP3, SIGMAR1, GAL, GALR1, GALR2, GALR3* | 40 | 169567 |
| Genes identified from recent GWAS | *UST, IL11, IL6, SLC27A1, RGL1, PLXDC2, ZNF326, MNX1, CDH17, FN1, ASTN1, NEDD4L, LOC100505738, TNFRSF10B, FKBP1A, NSFL1C, LRCH1, MYO10, ENOX1, KCNH5 , GMPR, ITGA11 , FLJ41603, NDFIP2, PIEZO2, RFPL4A, KCTD15, MIR548D2, PCDH1, GRIK3, SLC6A2, GRIA4, TBX21, LEP, GRIK1, HTR1A, HTR3A, UBE3C , BMP7 , RORA, ASIC2, ANKS1B, LRFN5, IQSEC1, CADPS, NPHP3, ACAD11, CFAP61, PRDM2, SLC37A1, IGFBP3 , SVIL, ARNTL, EIF4A1P1, NOL4, GOLGB1, SUMO1P1, LINC00955, MGST2, USPL1, HCLS1, YTHDC2, PSAT1, TLE4, LHFPL3, LARGE, SEMA5A, PDE9A, LRCH1, PDE9A, EPHB1 , AK090788andPDE10, ADAMTSL1, EDN1, EFNA5, EPHA5* | 89 | 421625 |

**Table SM5**. Functional annotation of rare variants

| Level-Score | Description |
| --- | --- |
| First1 | 1. Being reported in HGMD database |
|  | 1. Conservative |
|  | 1. Minor allele frequency lower than 0.001 in 1000genome or lower than .01 in the investigated control group |
|  | 1. Minor allele frequency lower than 0.01 in ESP6500 |
|  | 1. Called by at least one variant caller and there was no indels or homologous sequence nearby; besides, less than 50% of the samples have low genotyping-quality at this SNV |
|  | 1. Aligned to one specific region of the genome |
|  | 1. Minor allele frequency lower than 0.005 in Gene sky Database (exome sequencing data of 220 healthy Chinese samples) |
| First2 | 1. Minor allele frequency lower than 0.001 in 1000genome or lower than 0.01 in the investigated control group |
|  | 1. Minor allele frequency lower than 0.01 in ESP6500 |
|  | 1. Called by at least one variant caller and there was no indels or homologous sequence nearby; besides, less than 50% of the samples have low genotyping-quality at this SNV |
|  | 1. Aligned to one specific region of the genome |
|  | 1. Minor allele frequency lower than 0.005 in Genesky Database (exome sequencing data of 220 healthy Chinese samples) |
| Second | 1. Called by at least one variant caller and there was no indels or homologous sequence nearby; besides, less than 50% of the samples have low genotyping-quality at this SNV; 2. Aligned regions in the genome less than three |
|  | 1. Minor allele frequency lower than 0.001 in 1000genome or lower than 0.01 in the investigated control group |
|  |  |
| Third | The rest of SNVs. |

**Methods 2. Validating genotyping for top SNPs**

Thirty-one SNP genotypes were validated by re-genotyping in 506 controls and 776 patients with MDD for treatment response, which were randomly selected from the sequenced samples, only two SNPs failed to pass the pre-setted rate of accuracy in top signals for case-control analysis (removed and not reported). The genomic DNA was extracted using a Wizard Genomic DNA Purification Kit (Promega, Madison, Wisconsin, USA) per the product instruction. A multiplex PCR-ligase detection reaction method was used for genotyping. For each SNP, different fluorescent labels of allele-specific oligonucleotide probe pairs distinguished the alleles. Different extended lengths at 3' end further distinguished different SNPs. The products were separated and detected by capillary electrophoresis in an ABI3730XL sequencer. Raw data were analyzed according to the information obtained for the labeling dye color and fragment size of the allele-specific ligation-PCR product by GeneMapper v5.0.

1. Ivanov, S.V. & Samushiya, M.A. Agomelatine in the treatment of depressive disorders in clinical practice: multicenter observational CHRONOS study. *Neuropsychiatr Dis Treat* **10**, 631-9 (2014).
